# Regulation of the Sae Two-Component System by Branched-Chain Fatty Acids in Staphylococcus aureus

**DOI:** 10.1128/mbio.01472-22

**Published:** 2022-09-22

**Authors:** Augustus Pendleton, Won-Sik Yeo, Shahad Alqahtani, Dennis A. DiMaggio, Carl J. Stone, Zhaotao Li, Vineet K. Singh, Christopher P. Montgomery, Taeok Bae, Shaun R. Brinsmade

**Affiliations:** a Department of Biology, Georgetown University, Washington, DC, USA; b Center for Microbial Pathogenesis, Abigail Wexner Institute at Nationwide Children’s Hospital, Columbus, Ohio, USA; c Department of Microbiology and Immunology, A.T. Still University of Health Sciences, Kirksville, Missouri, USA; d Department of Pediatrics, College of Medicine, The Ohio State University, Columbus, Ohio, USA; e Department of Microbiology and Immunology, Indiana University School of Medicine-Northwest, Gary, Indiana, USA; Institut Pasteur

**Keywords:** MRSA, *Staphylococcus aureus*, SaeRS, virulence, membrane, branched-chain fatty acids, CodY, fatty acids, membranes, two-component regulatory systems, virulence regulation

## Abstract

Staphylococcus aureus is a ubiquitous Gram-positive bacterium and an opportunistic human pathogen. S. aureus pathogenesis relies on a complex network of regulatory factors that adjust gene expression. Two important factors in this network are CodY, a repressor protein responsive to nutrient availability, and the SaeRS two-component system (TCS), which responds to neutrophil-produced factors. Our previous work revealed that CodY regulates the secretion of many toxins indirectly via Sae through an unknown mechanism. We report that disruption of *codY* results in increased levels of phosphorylated SaeR (SaeR~P) and that *codY* mutant cell membranes contain a higher percentage of branched-chain fatty acids (BCFAs) than do wild-type membranes, prompting us to hypothesize that changes to membrane composition modulate the activity of the SaeS sensor kinase. Disrupting the *lpdA* gene encoding dihydrolipoyl dehydrogenase, which is critical for BCFA synthesis, significantly reduced the abundance of SaeR, phosphorylated SaeR, and BCFAs in the membrane, resulting in reduced toxin production and attenuated virulence. Lower SaeR levels could be explained in part by reduced stability. Sae activity in the *lpdA* mutant could be complemented genetically and chemically with exogenous short- or full-length BCFAs. Intriguingly, lack of *lpdA* also alters the activity of other TCSs, suggesting a specific BCFA requirement managing the basal activity of multiple TCSs. These results reveal a novel method of posttranscriptional virulence regulation via BCFA synthesis, potentially linking CodY activity to multiple virulence regulators in S. aureus.

## INTRODUCTION

Two-component systems (TCSs) are the predominant signal transduction system in bacteria for monitoring their intracellular and extracellular environments (1). Comprised of a membrane histidine kinase (HK) that becomes phosphorylated in response to a specific signal and its cognate response regulator (RR), which propagates the signal via phosphorylation and triggers the cellular response by controlling gene expression, TCSs facilitate nimble responses to chemical, biological, or physiological insults (1). Most bacterial species relevant to human health encode multiple TCSs controlling physiology and pathogenesis (2). For instance, the ubiquitous Gram-positive bacterium Staphylococcus aureus encodes 15 HK-RR pairs; an additional TCS (WalRK) is essential (3, 4). S. aureus exists both as a commensal of human nares and as a devastating pathogen responsible for hundreds of thousands of hospitalizations per year (5). Of these hospitalizations, approximately half are skin and soft tissue infections (SSTIs), and over 60% are linked to methicillin-resistant S. aureus (MRSA), which worsens patient outcomes and increases hospitalization costs (5). S. aureus relies on a finely tuned network of ligand-binding transcription factors, small RNAs (sRNAs), and TCSs to switch from a commensal to pathogenic lifestyle; these same regulatory systems control the toxin production, immune evasion, and biofilm formation which make S. aureus such a dangerous pathogen (6). These regulatory mechanisms represent potentially effective drug targets to address rising antibiotic resistance and improve clinical outcomes of MRSA and other bacterial infections.

CodY is a global regulator of metabolism and pathogenesis in low-G+C Gram-positive bacteria like S. aureus (7). S. aureus CodY binds branched-chain amino acids (BCAAs) (leucine, isoleucine, and valine) and GTP (8). When bound to these corepressors (predominantly isoleucine), CodY binds site-specific DNA sequences with the consensus motif AATTTTCWGAAAATT and represses the transcription of the majority of its targets (8–10). Direct metabolic targets of CodY include the *ilv* operon responsible for BCAA synthesis in addition to numerous genes responsible for amino acid synthesis, import, and degradation (8, 11).

CodY also plays a central role in regulating virulence in S. aureus, and deletion of *codY* leads to hypervirulence in SSTIs (12). CodY represses some virulence factor genes directly, including those for Panton-Valentine leukocidin (*lukSF-PV*) and capsular polysaccharide (*cap*) (8, 12, 13). However, much of CodY’s influence on virulence is indirect through control of two major virulence regulatory systems—the Agr quorum sensing system and the focus of this study, the SaeRS TCS (9, 14, 15). The Sae TCS is an important virulence regulator which activates the expression of over 20 virulence factors, including α-hemolysin (*hla*) and β-hemolysins (*hlb*), bicomponent pore-forming toxins including γ-hemolysin (*hlgABC*) and Panton-Valentine leukocidin (*lukSF*), toxic shock syndrome toxin 1 (*tst*), coagulase (*coa*), and nuclease (*nuc*) (14, 16).

The *sae* locus consists of four genes (*saeP*, *saeQ*, *saeR*, and *saeS*), whose expression is driven from two promoters: a constitutive promoter (P3) that provides the basal levels of the SaeR and SaeS proteins required for sensing and responding to changes in environmental conditions and an autoregulated promoter (P1) that is activated by the SaeR protein through protein phosphorylation when the bacterium is exposed to specific signals such as human neutrophil peptides (HNPs) (14, 17–19). SaeS is a sensor histidine kinase, with two N-terminal transmembrane domains and a cytoplasmic C-terminal domain responsible for ATP binding, autokinase activity, and phosphotransferase activity (20, 21). SaeS is strongly activated by HNPs as well as calprotectin, β-lactam antibiotics, and hydrogen peroxide (19, 22, 23). SaeS activation also increases in later growth phases in an *agr-*independent manner (18). Notably, SaeS is a member of the intramembrane family of sensor kinases; as such, the extracellular portion of SaeS consists only of a 9-amino-acid linker peptide that connects its two transmembrane domains and constrains its activity (20, 24, 25). While mutations in this linker peptide can affect activation, signal perception likely occurs within the transmembrane domains (20). Two additional proteins, SaeP and SaeQ, form a ternary complex with SaeS to stimulate SaeS’s phosphatase activity to prevent overexpression of SaeR targets (26). CodY represses *sae* expression directly by binding the upstream region of the *sae*P1 promoter to potentially hinder binding of SaeR to the P1 promoter and indirectly by keeping Agr activity low (27). However, P1 drives the production of both activator proteins for Sae-dependent genes (SaeRS) and inhibitor proteins (SaePQ). This poses a conundrum—how can CodY effectively control Sae and regulate virulence factor production solely through the P1 promoter? Under conditions where CodY activity is reduced, production of SaePQ can prevent Sae activity and virulence expression from becoming unlimited (26, 28). However, *saeRS* transcription, Sae activity, and target gene expression are poorly correlated, complicating a simple transcriptional model of CodY repression via the P1 promoter (21, 27). Accordingly, Sae activity may be modulated posttranscriptionally via alternative CodY-dependent pathways.

This study investigates the mechanisms required for CodY-dependent repression of Sae activity and identifies branched-chain fatty acid (BCFA) metabolism and membrane homeostasis as novel regulators of virulence factor production. Previous work in Bacillus subtilis and S. aureus has highlighted the role of CodY in branched-chain amino acid synthesis and import, and these pathways both result in conversion to their respective keto acids that can serve as precursors for fatty acid synthesis (8, 29, 30). These branched-chain fatty acids (BCFAs) comprise the majority of membrane phospholipids in S. aureus (31). Compared to the parent strain, we show that *codY* mutant membranes are rich in branched-chain fatty acids, and we demonstrate genetically and biochemically that membranes lacking BCFAs and, in particular, 15:0 anteiso-BCFAs result in major impacts to sensor kinase activation, including the major virulence regulator SaeS. We also demonstrate that sufficient levels of branched-chain fatty acids are essential for pathogenesis in a mouse model of SSTI.

## RESULTS

### SSTI hypervirulence in a *codY* mutant requires both *sae* and *hla*.

**FIG 1:**
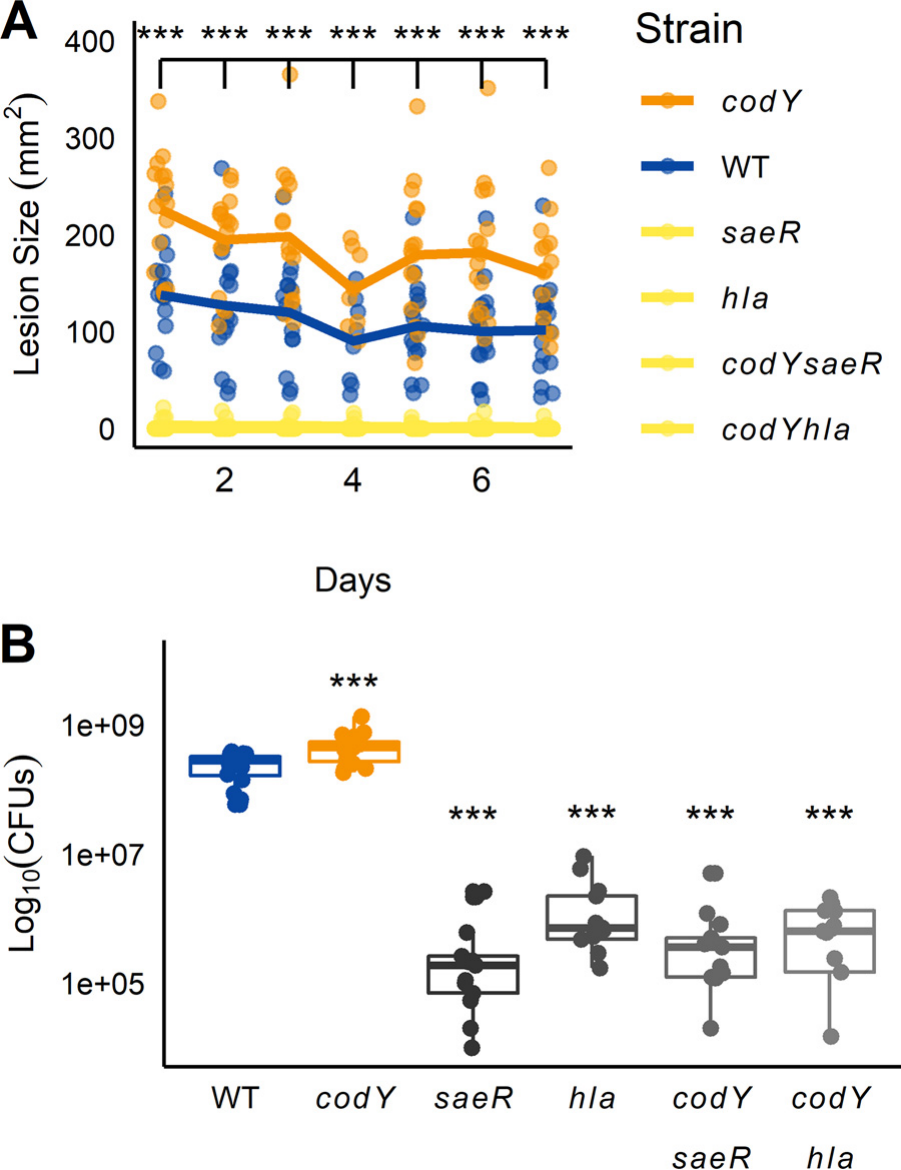
Hypervirulence of a *codY* null mutant requires *saeRS* and *hla*. (A) BALB/c mice were infected subcutaneously with one of six S. aureus strains, and the size of dermonecrotic lesions was quantified for 7 days postinfection. Data for *saeR*, *codY saeR*, *hla*, and *codY hla* strains were indistinguishable at this scale and not statistically significantly different from each other and so are plotted using the same color. Lines represent means from 12 mice ± standard deviation. ***, *P* < 0.001, two-way ANOVA with repeated measures with Tukey *post hoc* test relative to wildtype (WT) *each* day. (B) Lesion CFU count 7 days postinfection. ***, *P* < 0.001, ANOVA with Tukey *post hoc* test relative to WT.

Previous work showed that deleting the *codY* gene in a USA300 clone of S. aureus resulted in a hypervirulent phenotype during skin infection, producing a larger maximum area of dermonecrosis than that of the isogenic parent strain (12). While the genes coding for Panton-Valentine leukocidin (*lukSF-*PVL) were overexpressed in the *codY* mutant, deleting *lukSF* did not attenuate virulence in that background. However, the contribution of Panton-Valentine leukocidin (*lukSF-*PVL) to virulence in mice is unclear, as murine C5aR binds PVL poorly (32). Moderate overexpression of *saeRS*—a classical regulator of virulence in S. aureus—was also observed in the *codY* mutant, as was that of the Sae-dependent gene *hla* (9, 33). Thus, we hypothesized that the *codY* hypervirulence phenotype may be due to altered Sae-dependent gene expression. To test this hypothesis, we challenged female BALB/c mice with either our wild-type (WT) USA300 strain LAC, the *codY* mutant, a *saeR* mutant, or a *saeR codY* double mutant and tracked lesion size over time (34, 35). As expected, mice challenged with the *codY* mutant consistently showed increased lesion size relative to LAC within the first week of infection (Fig. 1A). In contrast, the *saeR* mutant and the *codY saeR* double mutant did not induce dermonecrosis, though they did establish subcutaneous abscesses. Additionally, *saeR* and *codY saeR* lesions contained fewer CFU than did those of either the WT or the *codY* mutant (Fig. 1B). Given the known role of α-hemolysin in dermonecrosis and the fact that the Sae TCS is an important modulator of *hla* gene expression (35, 36), we then wondered if the hypervirulence was due to increased *hla* expression in the *codY* mutant. Dermonecrosis was significantly reduced in the *hla* single mutant and the *hla codY* double mutant, and CFU counts were reduced (Fig. 1). Taken together, our data indicate that the hypervirulence phenotype observed for mice challenged with *codY* mutant cells requires the Sae TCS and *hla*.

### Disruption of *codY* promotes Sae activity, independent of positive autoregulation by the P1 promoter.

**FIG 2:**
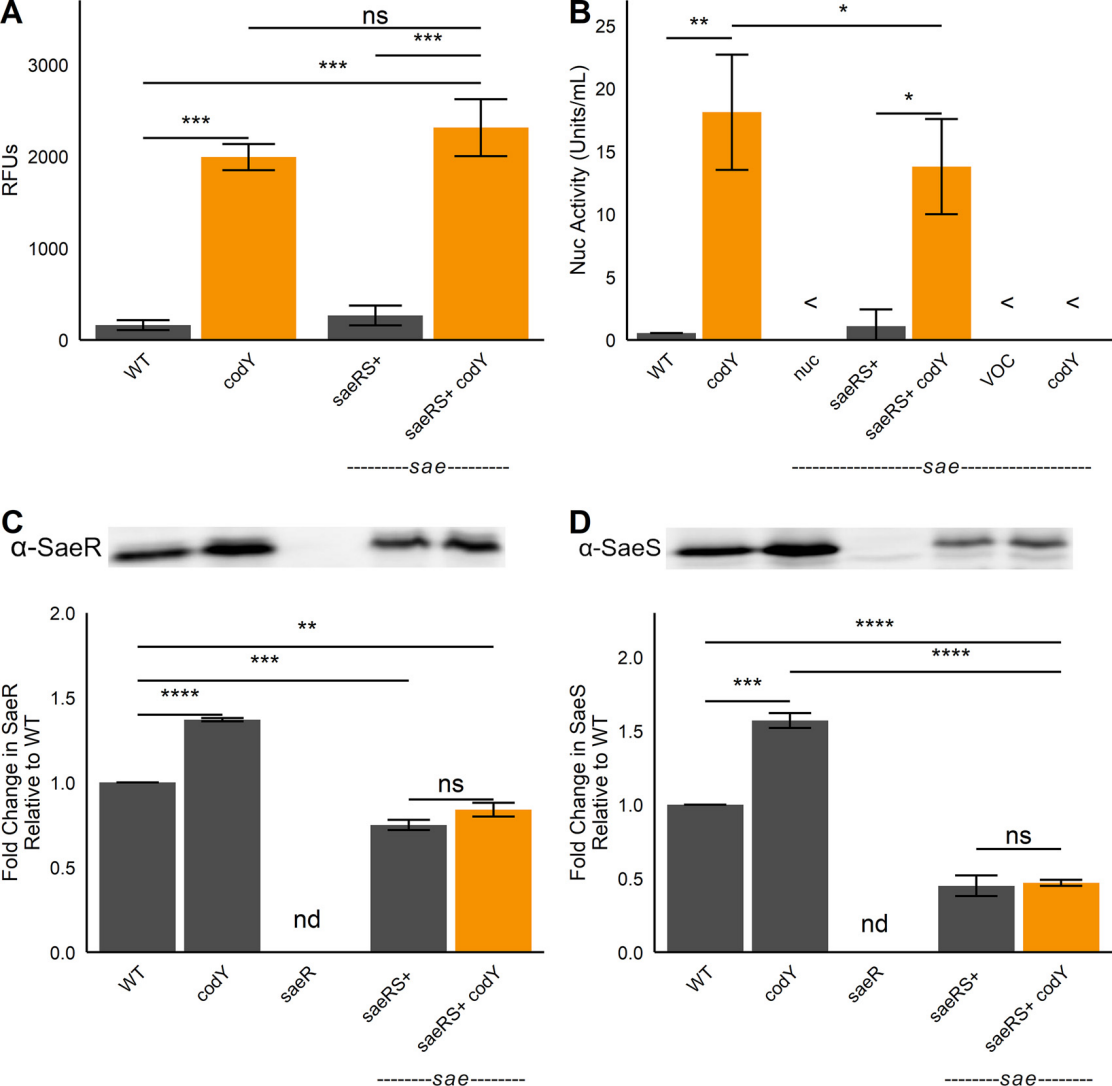
SaeR and SaeS are necessary and sufficient to upregulate *nuc* when *codY* is knocked out. The indicated LAC strains were grown to exponential phase in tryptic soy broth (TSB) at which time *nuc-gfp* promoter activity (A) or secreted nuclease activity (B) was measured. The intracellular abundances of SaeR (C) and SaeS (D) were detected in the indicated strains by Western blot analysis using polyclonal antibodies raised against S. aureus proteins (top panel). Protein levels were normalized via BCA protein assay, and loading consistency was verified using Coomassie blue stains (see Fig. S5 in the supplemental material). Densitometric quantification (bottom panels) was performed using ImageJ software (National Institutes of Health). *, *P* < 0.05; **, *P* < 0.01; ***, *P* < 0.001; ****, *P* < 0.0001, Tukey multiple-comparison test after analysis of variance (ANOVA). <, indistinguishable from blank; ns, not significant; nd, not detected.

We previously showed that CodY controls virulence factor production in part via the Sae TCS (9, 27). Those studies focused on transcriptional control of the *sae* locus, and our results revealed that CodY directly and indirectly adjusts *sae* expression by repressing the *sae*P1 promoter (27). However, there is little correlation between the expression level of the *saeR* and *saeS* genes and that of SaeR target genes like *nuc*, *hla*, *hlb*, and *eap* (21). Rather, it is the activity of the SaeS kinase and DNA-binding activity of SaeR that is key, not protein levels *per se*. Therefore, we expect that the P1 promoter is dispensable for the activation of Sae when CodY activity is reduced or eliminated. To evaluate the significance of the P1 promoter, we deleted the *sae* locus and complemented the strain with an integrated *saeRS* construct under the control of the native *sae*P3 promoter. We then introduced a *nuc-gfp* reporter plasmid into this strain and its *codY* mutant derivative, respectively, and compared promoter activities in these strains to those in the WT and the *codY* single mutant. Levels of *nuc-gfp* reporter activity were essentially identical in the WT and the *saeRS*-complemented strain. As expected, reporter activity increased 12-fold in the *codY* mutant. Notably, reporter activity in the *codY saeRS*-complemented strain was similarly upregulated (Fig. 2A). Similar results were also obtained when we measured secreted nuclease activity in medium conditioned by the same strains (Fig. 2B). We determined that the increased activity did not require an associated increase in SaeR and SaeS protein levels (Fig. 2C and D); the modest increases seen in the *codY* mutant likely result from readthrough from the *sae*P1 promoter through positive autoregulation when CodY activity is reduced (37). Taken together, our data indicate that the known direct and indirect regulation at *sae*P1 by CodY is dispensable for controlling Sae-dependent genes, and CodY regulation does not control the levels of the SaeRS proteins.

10.1128/mbio.01472-22.5 FIG S5 Representative Coomassie blue-stained loading control for SaeR and SaeS Western blot analyses (Fig. 2). Protein concentration of total cell lysates was determined using the BCA assay, and gels were run in duplicate for Western blotting and Coomassie blue staining. Download FIG S5, TIF file, 2.6 MB. Copyright © 2022 Pendleton et al. 2022 Pendleton et al. https://creativecommons.org/licenses/by/4.0/ This content is distributed under the terms of the Creative Commons Attribution 4.0 International license.

### Lack of CodY increases SaeR**~**P levels *in vivo*.

**FIG 3:**
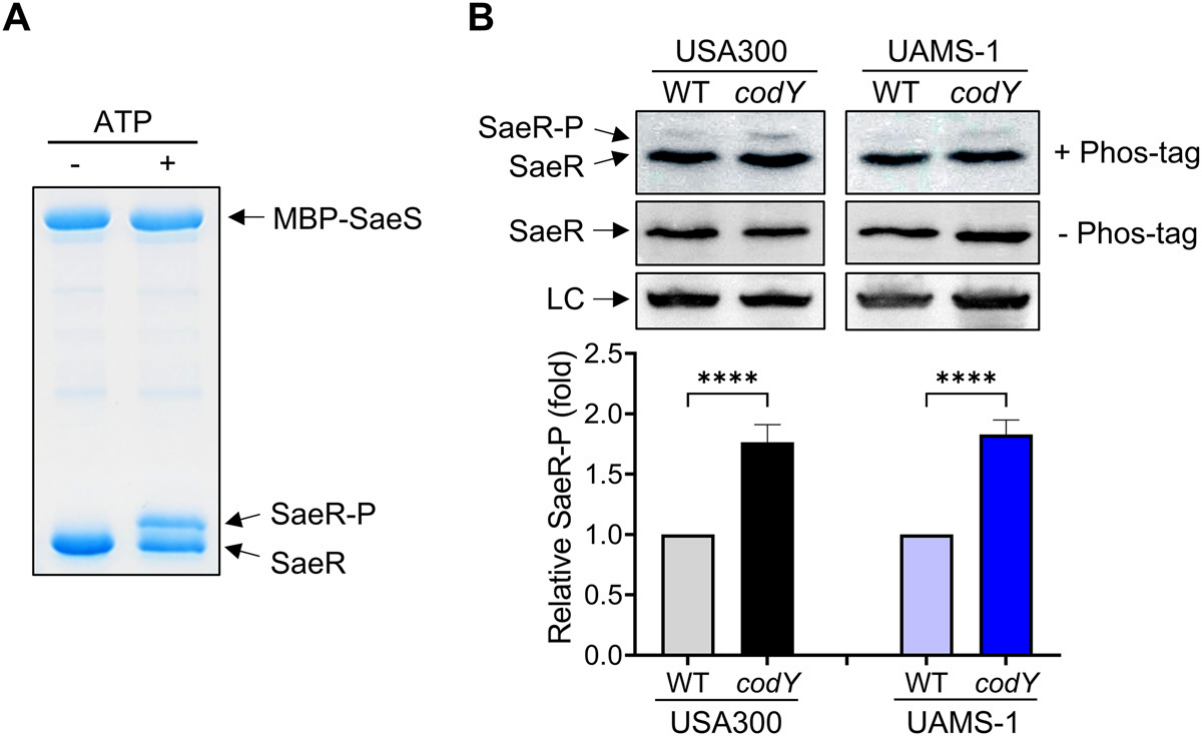
Disruption of *codY* increases SaeR~P levels *in vivo*. (A) *In vitro* phosphorylation of SaeR by SaeS. Purified proteins were incubated in the presence or absence of ATP. SaeS, SaeR, and SaeR~P were separated on Phos-tag polyacrylamide gels and visualized by Coomassie blue staining. (B) Phos-tag Western blot (top) or Western blot (middle and bottom) analysis of crude extracts prepared from USA300 or UAMS-1 strains (WT and *codY*) grown in TSB medium for 5 h using antibodies recognizing SaeR. LC, loading control. The graph depicts levels of SaeR~P relative to SaeR~P from the wild type. To avoid splicing gel images, the *sae* negative control (run on the same gel) appears in Fig. 7A. All data are representative of at least three independent experiments, which produced similar results. One-way ANOVA with Tukey correction was used for statistical analysis comparing SaeR~P levels. *n* > 3. ****, *P* < 0.0001.

The direct output of a two-component system in relation to gene transcription depends on the levels of the phosphorylated response regulator that is regulated by its cognate sensor histidine kinase. Therefore, we reasoned that the increased *nuc* promoter activity observed in the *codY* mutant might be due to altered SaeS kinase activity. To test this notion, we examined the amount of phosphorylated SaeR (SaeR~P) by using Phos-tag gels and Western blotting with antibodies recognizing SaeR (38, 39). We first carried out an *in vitro* phosphorylation assay with purified proteins by incubating polyhistidine-tagged SaeR (SaeR-His_6_) and maltose-binding protein-tagged SaeS (MBP-SaeS) in the absence or presence of 1 mM ATP, and the mixtures were subjected to Phos-tag gel electrophoresis to separate SaeR and SaeR~P. As expected, SaeR~P migrated slower than unphosphorylated SaeR (Fig. 3A). Next, we examined the amount of SaeR~P in crude extracts from wild-type and *codY* mutant cells and determined that the levels of SaeR~P were higher in the *codY* mutant than in the wild type (Fig. 3B). The levels of SaeR~P were also increased in the USA200, methicillin-susceptible, osteomyelitis isolate (UAMS-1) *codY* mutant (Fig. 3B), which is consistent with the previous result that the *nuc* activity was increased in the UAMS-1 *codY* mutant (9). Neither SaeR nor SaeR~P was detected in the *sae* mutant (Fig. 3B). Increased SaeR~P was also observed with *codY* mutant membrane vesicles, which were used as the source of SaeS in a kinase assay *in vitro* (see Fig. S1 in the supplemental material); note that the SaeR~P was normalized by the level of the SaeS protein in the membranes determined by Western blot analysis. These results agree with the notion that lack of CodY renders SaeS more active, resulting in an increase of the SaeR~P abundance.

10.1128/mbio.01472-22.1 FIG S1 Lack of CodY results in increased SaeS kinase activity. (A) Levels of SaeR~P after incubation of recombinant His_6_-SaeR with wild-type (WT) or *codY* mutant membrane vesicles from USA300 or UAMS-1 in the presence of 0.1 mM [γ-^32^P]ATP as a source of ATP. Levels of SaeR~P were normalized by the level of SaeS protein in the membrane vesicles. (B and C) Quantification of the kinase assays shown in panel A. The graph depicts the levels of SaeR~P at the indicated time point relative to SaeR~P at time 30 s in the wild-type membranes. All data correspond to the mean ± SEM from three independent experiments. For statistical analyses, a two-way ANOVA with Šidák correction was used for statistical analysis comparing each SaeR~P level between the wild type and the *codY* mutant at the indicated time points. *, *P* < 0.05; **, *P* < 0.01; ***, *P* < 0.001; ****, *P* < 0.0001. Download FIG S1, TIF file, 0.6 MB. Copyright © 2022 Pendleton et al. 2022 Pendleton et al. https://creativecommons.org/licenses/by/4.0/ This content is distributed under the terms of the Creative Commons Attribution 4.0 International license.

### Changes in CodY activity alter membrane composition in S. aureus.

**FIG 4:**
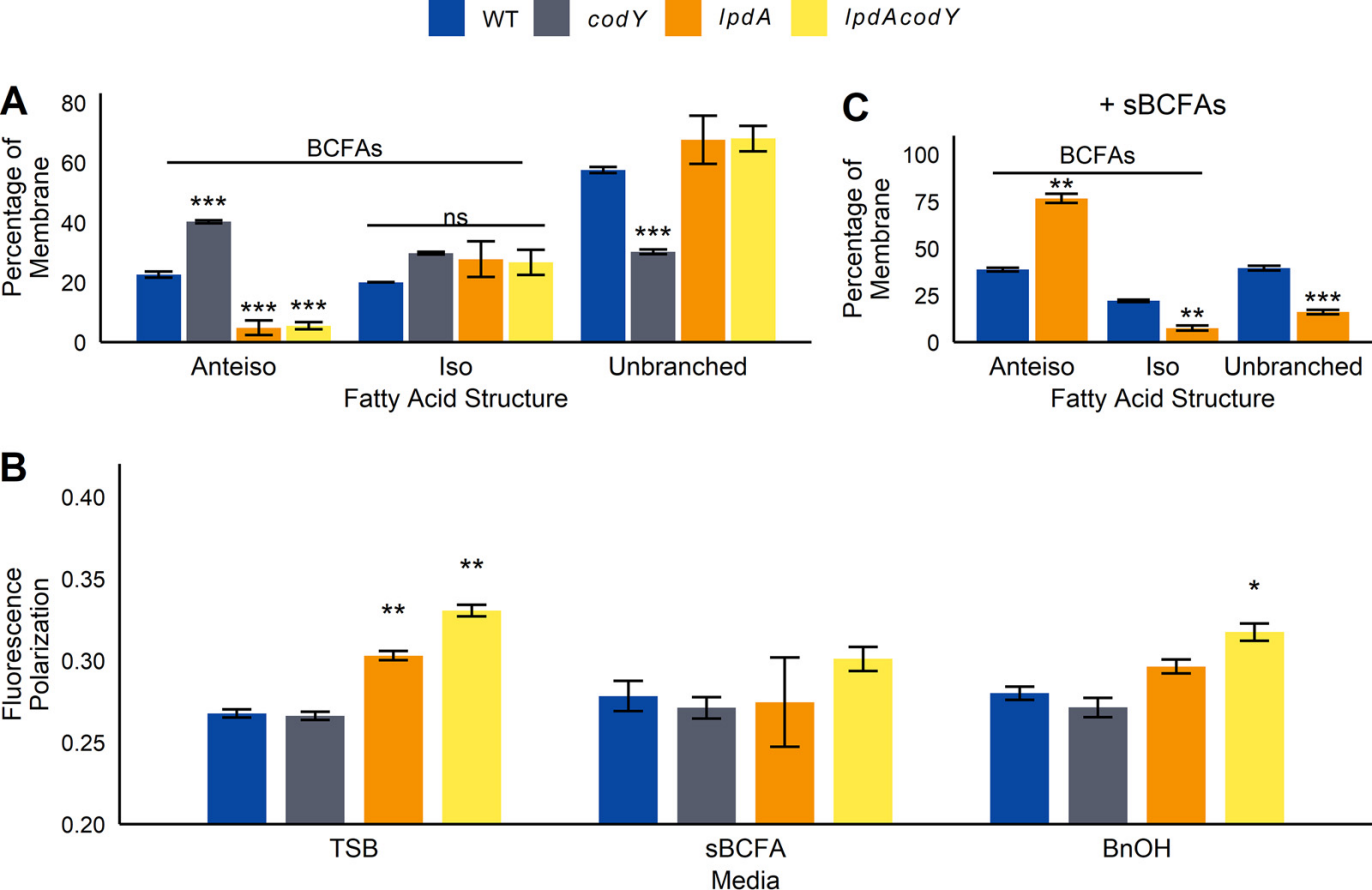
Disruption of *codY* or *lpdA* affects membrane fatty acid composition. (A) Fatty acid composition was measured in exponential-phase cells using gas chromatographic analysis of fatty acid methyl esters (GC-FAME) (*n* = 3). (B) Membrane polarization was measured in exponential-phase cells. Membrane polarization is inversely correlated with membrane fluidity (*n* = 4). (C) GC-FAME for strains grown to exponential phase supplemented with 0.5 mM sBCFAs. Because samples were prepared and analyzed separately, comparisons between panels A and C are not made (*n* = 3). For all panels, bars represent mean ± standard deviation. *, *P* < 0.05; **, *P* < 0.01; ***, *P* < 0.001; ns, not significant compared to wild type under that condition, by either ANOVA with Tukey’s *post hoc* test (A) or ANOVA followed by Student’s *t* tests with Bonferroni correction (B and C).

As an intramembrane histidine kinase, SaeS contains two transmembrane domains whose conformation may be affected by changes in the membrane environment, resulting in altered enzymatic activity (20, 40). Reducing CodY activity in Bacillus subtilis and S. aureus results in derepression of the BCAA biosynthetic genes and increases BCAA synthesis, particularly that of (iso)leucine and valine (9, 41, 42). BCAA import is also increased (9, 43). The resulting branched-chain α-keto acids can be diverted to pantothenate and branched-chain fatty acid synthesis; the latter can be used to adjust membrane fluidity and integrity (44). We reasoned that an altered membrane composition and/or fluidity in the *codY* mutant may contribute to the increased SaeS kinase activity measured *in vitro*. To determine the impact of CodY on membrane composition, we subjected wild-type and *codY* strains to gas chromatographic analysis of fatty acid methyl esters (GC-FAME), which measures total fatty acid content of the membrane. We included a mutant with a transposon insertion in *lpdA* (dihydrolipoyl dehydrogenase subunit of the branched-chain α-keto acid dehydrogenase [BKDH] complex; catalyzes the synthesis of acyl coenzyme As [acyl-CoAs] for BCFA synthesis [31]), as a control for reduced BCFA content. The *codY* cell membranes had significantly higher levels of anteiso- and iso-structured BCFAs than did wild-type cell membranes and a corresponding decrease in unbranched fatty acids (Fig. 4A). An increase in anteiso-BCFAs was also observed in a Bacillus subtilis *codY* mutant, and while total BCFAs did not significantly change in Listeria monocytogenes, BCFA chain length did vary in a *codY* mutant (Fig. S2 and Table S1). No changes were observed in Clostridioides difficile (Fig. S2). As expected, the *lpdA* and *lpdA codY* mutant membranes contained significantly less anteiso-BCFA content than did the wild type and more unbranched fatty acids, though surprisingly similar levels of iso-BCFAs (Fig. 4A).

10.1128/mbio.01472-22.2 FIG S2 Deletion of *codY* alters membrane composition in (A) *S. aureus* and (B) B. subtilis but not (C) L. monocytogenes or (D-E) C. difficile. Strains were grown to exponential phase before membrane composition was analyzed using gas chromatographic analysis of fatty acid methyl esters (GC-FAME). “Other” fatty acids include hydroxy-, cyclopropane-, and dimethyl acetal-substituted fatty acids. *, *P* < 0.05; **, *P* < 0.01; ***, *P* < 0.001; ns, nonsignificant. Student’s *t* test between strains for each fatty acid type with Bonferroni correction. Download FIG S2, TIF file, 0.3 MB. Copyright © 2022 Pendleton et al. 2022 Pendleton et al. https://creativecommons.org/licenses/by/4.0/ This content is distributed under the terms of the Creative Commons Attribution 4.0 International license.

10.1128/mbio.01472-22.6 TABLE S1 Even- and odd-chain iso-BCFAs respond to *codY* deletion in L. monocytogenes differently. Percent difference represents difference in *codY* strain compared to wild type ± standard deviation for each fatty acid as determined by GC-FAME (*n* = 3). While no significant differences in iso-BCFA content were found between wild-type and *codY* strains (Fig. S2C), even-chain BCFAs consistently increased in the *codY* mutant while odd-chain BCFAs consistently decreased. Download Table S1, DOCX file, 0.01 MB. Copyright © 2022 Pendleton et al. 2022 Pendleton et al. https://creativecommons.org/licenses/by/4.0/ This content is distributed under the terms of the Creative Commons Attribution 4.0 International license.

BCFAs, especially 15:0 anteiso-fatty acids, increase membrane fluidity and are important for survival in S. aureus (45). Using membrane polarization, we determined membrane fluidity in *codY* and *lpdA* strains (Fig. 4B). In plain tryptic soy broth (TSB), *codY* membrane fluidity was unchanged, while both *lpdA* strains had significantly higher polarization (lower fluidity) than wild-type membranes. After supplementation with short, BFCA precursors isovaleric acid, isobutyric acid, and 2-methylbutyric acid (sBCFAs), membrane fluidity was not significantly different between wild-type and *codY*, *lpdA*, and *lpdA codY* mutant strains. Supplementing with the membrane fluidizer benzyl alcohol (BnOH) (46) also reduced differences in membrane polarization between the *lpdA* mutant and the wild type, though the *lpdA codY* mutant still had significantly higher polarization (Fig. 4B). Taken together, our data indicate that strains lacking *codY* have more BCFAs in the membrane but compensate to maintain wild-type levels of membrane fluidity, while *lpdA* strains have both fewer BCFAs and lower membrane fluidity.

### BCFAs are required for basal and stimulated SaeS activity.

**FIG 5:**
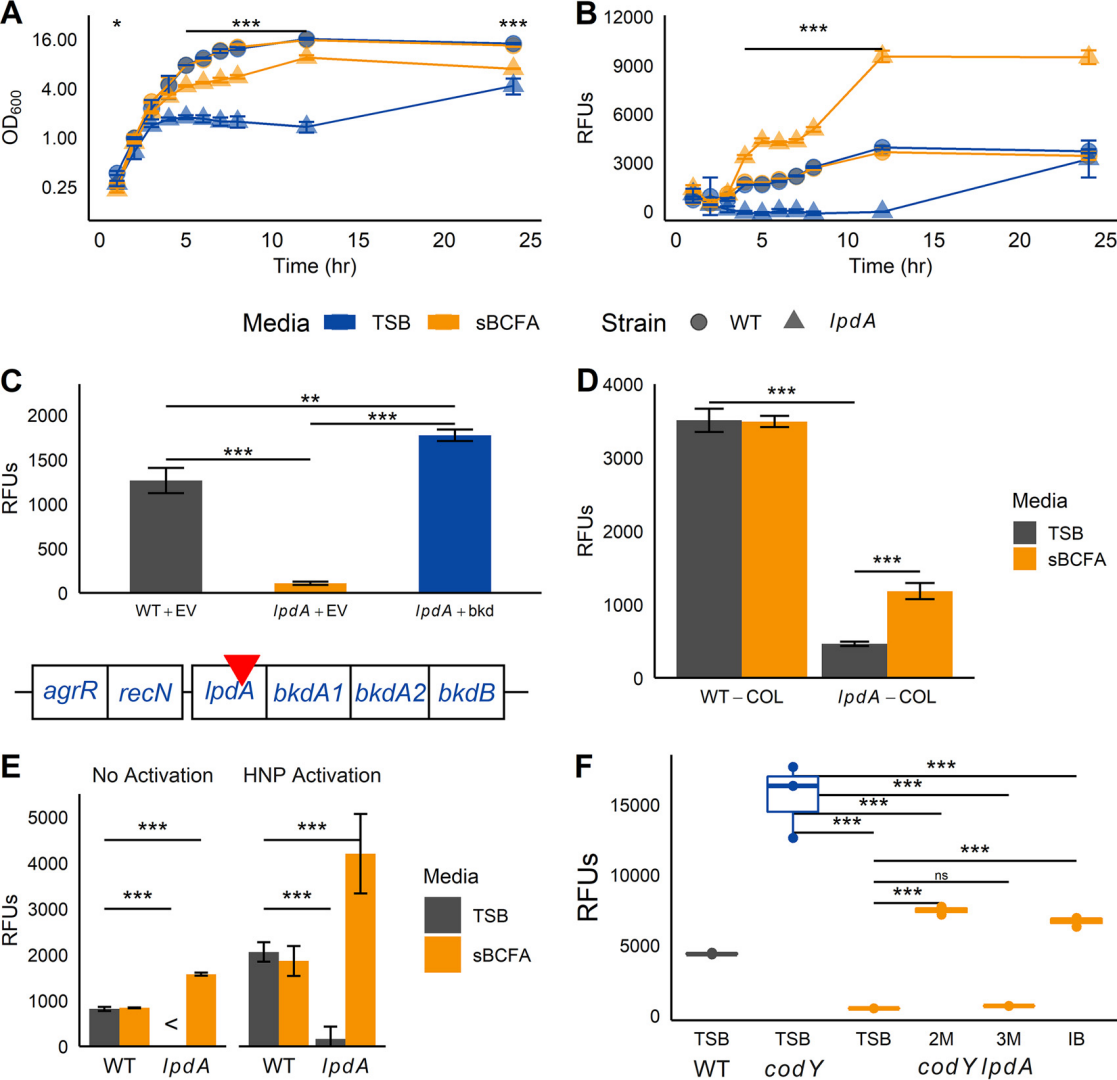
Defects in BCFA synthesis result in defects in Sae activation. (A and B) Wild-type (WT) and *lpdA* strains harboring *sae*P1*-gfp* reporter fusions were grown aerobically in TSB with or without 0.5 mM sBCFAs at 37°C for 24 h; at each hour, optical density (A) and relative fluorescence units (RFUs) (B) were measured. (C) Figure shows structure of the putative *bkd* operon, with transposon insertion indicated by the red inverted triangle. Wild-type and *lpdA* cells harboring a *sae*P1*-gfp* reporter fusion were complemented with either empty vector (EV) or the full *bkd* cluster (*bkd*) and grown for 5 h before GFP production was measured. (D) The USA300 *lpdA* transposon insertion was transduced into S. aureus COL, along with the *sae*P1*-gfp* reporter fusion. Cells were grown for 5 h before GFP production was measured. (E) Cells harboring *sae*P1*-gfp* reporter fusions were grown for 2 h before addition of 5 μg mL^−1^ HNP-1 (HNP activation) or carrier (15 mM Tris-Cl, pH 8, no HNP activation) and grown for an additional 2.5 h before cell GFP production was measured. (F) Cells harboring the *sae*P1*-gfp* reporter fusion were grown for 5 h in plain TSB or TSB supplemented with 0.5 mM 2-methylbutyric acid (2M), 3-methylbutyric acid (3M), or isobutyric acid (IB) before GFP production was measured. For all panels, symbols were as follows: *, *P* < 0.05; **, *P* < 0.01; ***, *P* < 0.001, determined using ANOVA with repeated measures and Tukey *post hoc* test (A and B) or ANOVA with Tukey *post hoc* test (C, D, E, and F); <, GFP fluorescence less than that of pure PBS used to resuspend the cells for measurement; ns, not significant. In panel 5A, asterisks indicate that the *lpdA* mutant is significantly different compared to WT in TSB at each time point.

**(i) Basal and induced Sae activation is reduced in *lpdA* mutants.** With the identification of an altered membrane composition in *codY* and *lpdA* mutant membranes, we next addressed the possibility that membrane BCFAs affect SaeR/S activity. To begin to answer this question, we measured *sae*P1*-gfp* promoter activity in the *lpdA* mutant. Compared to WT, growth of the *lpdA* mutant is impaired in TSB medium and is partially restored with sBCFA supplementation (Fig. 5A). Notably, *sae*P1*-gfp* promoter activity is severely reduced in the *lpdA* mutant compared to the WT between 4 and 12 h (Fig. 5B). Overexpression of *lpdA* and the downstream genes for the BKDH complex in the *lpdA* mutant increases *sae*P1-*gfp* promoter activity over that measured in WT cells (Fig. 5C). Interestingly, the same effect was achieved when we supplemented with sBCFAs, and this was correlated with a significantly higher percentage of anteiso-BCFAs in the membrane (Fig. 5B and Fig. 4C). Despite multiple attempts, we were not successful in constructing a UAMS-1 *lpdA* mutant. However, we found that the Sae activation defect was also apparent in an *lpdA* mutant of strain COL (47), demonstrating this is not a strain-specific phenomenon (Fig. 5D). Sae activation in response to HNP-1 stimulation was also significantly reduced in LAC *lpdA* cells compared to WT cells (Fig. 5E). In fact, sBCFAs (specifically 2-methylbutyric acid, the precursor to 15:0 anteiso-BCFAs, and isobutyric acid, the precursor to 14:0 iso-BCFAs) were required for Sae activation in a *codY lpdA* background, though they could not rescue Sae activation to levels observed in the *codY* mutant (Fig. 5F).

**FIG 6:**
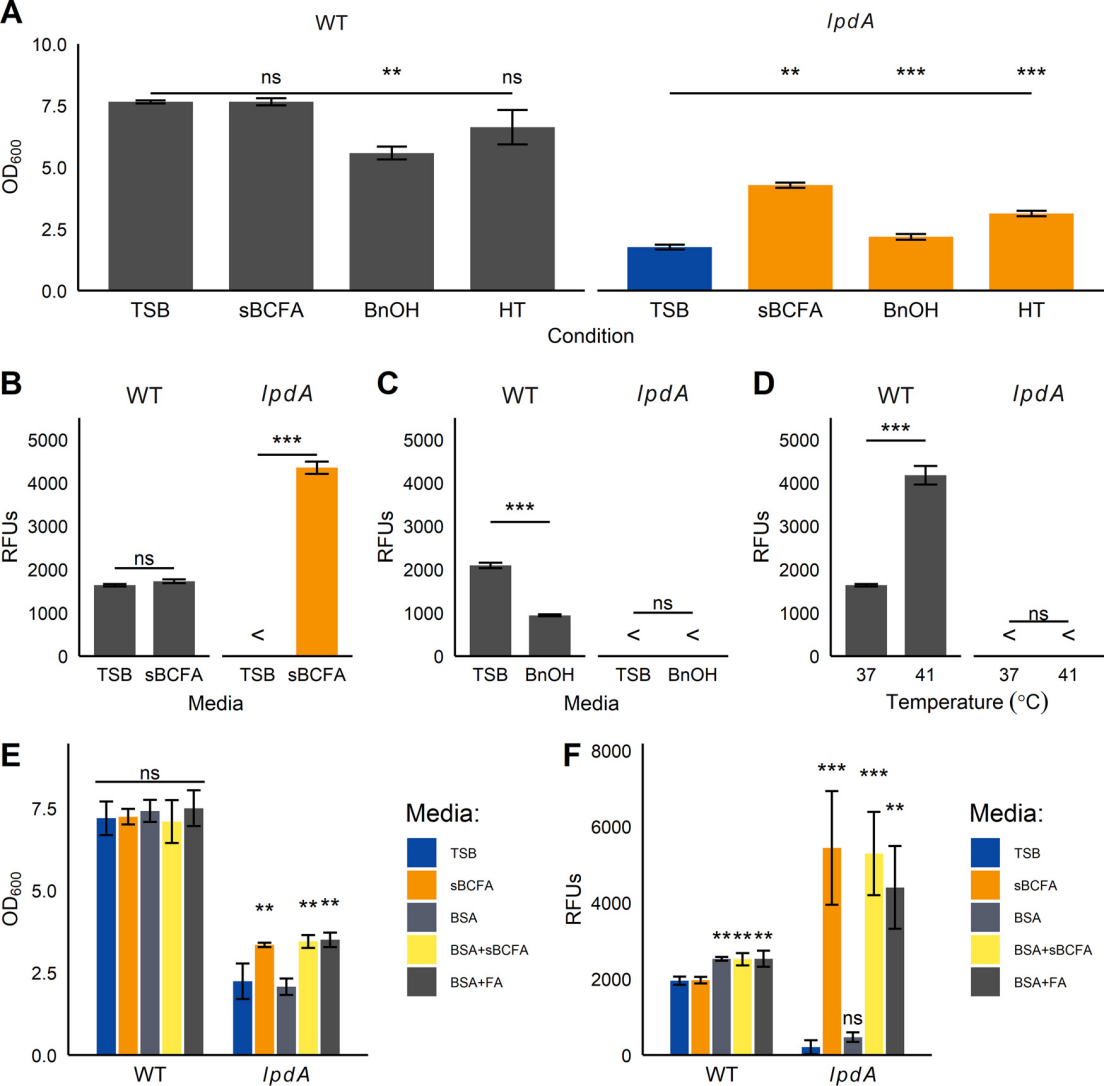
Branched-chain fatty acids are required for Sae activation. (A to D) Strains carrying *sae*P1*-gfp* reporter fusions were grown under three fluidizing conditions: 0.5 mM sBCFA supplementation (sBCFA), 0.1% benzyl alcohol (BnOH), or growth at 41°C (HT). After 5 h, optical density (OD) (A) and relative fluorescence units (RFUs) (B to D) were measured. (B) Fluorescence in sBCFA-supplemented medium, (C) Fluorescence in BnOH-supplemented medium, (D) Fluorescence at a higher temperature. <, fluorescence was undetectable compared to blank PBS. (E and F) Strains were grown in TSB with or without 0.5 mM sBCFAs, 10 mg mL^−1^ BSA, and 0.5 mM 15:0 anteiso-fatty acid (FA), before optical density (E) and relative fluorescence units (RFUs) (F) were measured. For all panels, bars represent mean from three trials ± standard deviation. ns, not significant; *, *P* < 0.05; **, *P* < 0.01; ***, *P* < 0.001, ANOVA with Tukey *post hoc* test. In panel A, significance is relative to the indicated strain in TSB.

**(ii) Fluidizing conditions improve growth but not Sae activation in *lpdA* mutants.** In principle, the reduction of SaeS kinase activity in the *lpdA* mutant could be due either to a decrease in membrane fluidity or to a specific requirement for BCFAs. To distinguish between these two possibilities, we measured Sae activation under three fluidizing conditions: supplementation with 0.5 mM sBCFAs, supplementation with 0.1% (vol/vol) benzyl alcohol, and growth at 41°C. After 5 h of growth in these media, *lpdA* cultures reached significantly higher optical density under all three fluidizing conditions (Fig. 6A). However, only the addition of sBCFAs resulted in significant increases in Sae activation for the *lpdA* mutant (Fig. 6B to D).

Previous work showed that disrupting fatty acid kinase (*fakA*) increases the pool of nonesterified straight-chain fatty acids (StrFAs) in the membrane and inhibits SaeS activity, resulting in decreased α-hemolysin production, increased protease production, and increased dermonecrosis (48–50). Removing straight-chain FAs with fatty acid-free bovine serum albumin (BSA) restores SaeS activity without affecting growth (49). This phenotype is conserved in our USA300 LAC strain (Fig. S3), and it is possible that the relative increase in straight-chain FAs observed in the *lpdA* mutant inhibits SaeS. To test whether straight-chain FAs are responsible for Sae defects in the *lpdA* mutant, we supplemented *lpdA* cells with BSA, sBCFAs, and full-length BCFAs. Supplementation with sBCFAs or 15:0 anteiso-BCFA improves both the growth and Sae activation of the *lpdA* mutant (Fig. 6E and F). However, BSA addition does not rescue Sae activation in the *lpdA* mutant, though it does increase Sae activation in the wild type (Fig. 6F).

10.1128/mbio.01472-22.3 FIG S3 Disruption of fatty acid kinase (*fakA*) inhibits Sae activation in USA300 LAC. Strains carrying a s*ae*P1*-gfp* reporter fusion responsive to Sae activation were grown for 5 h in TSB ± 0.5 mM sBCFAs, 10 mg mL^−1^ BSA, and 0.5 mM 15:0 anteiso-fatty acid (FA), before OD_600_ (A) and relative fluorescence units (RFUs) (B) were measured. RFUs are normalized by OD. Mean from three trials ± standard deviation. *, *P* < 0.05; **, *P* < 0.01; ***, *P* < 0.001, versus that strain in TSB; ns, not significant, two-way ANOVA followed by Tukey *post hoc* test. Download FIG S3, TIF file, 0.1 MB. Copyright © 2022 Pendleton et al. 2022 Pendleton et al. https://creativecommons.org/licenses/by/4.0/ This content is distributed under the terms of the Creative Commons Attribution 4.0 International license.

**FIG 7:**
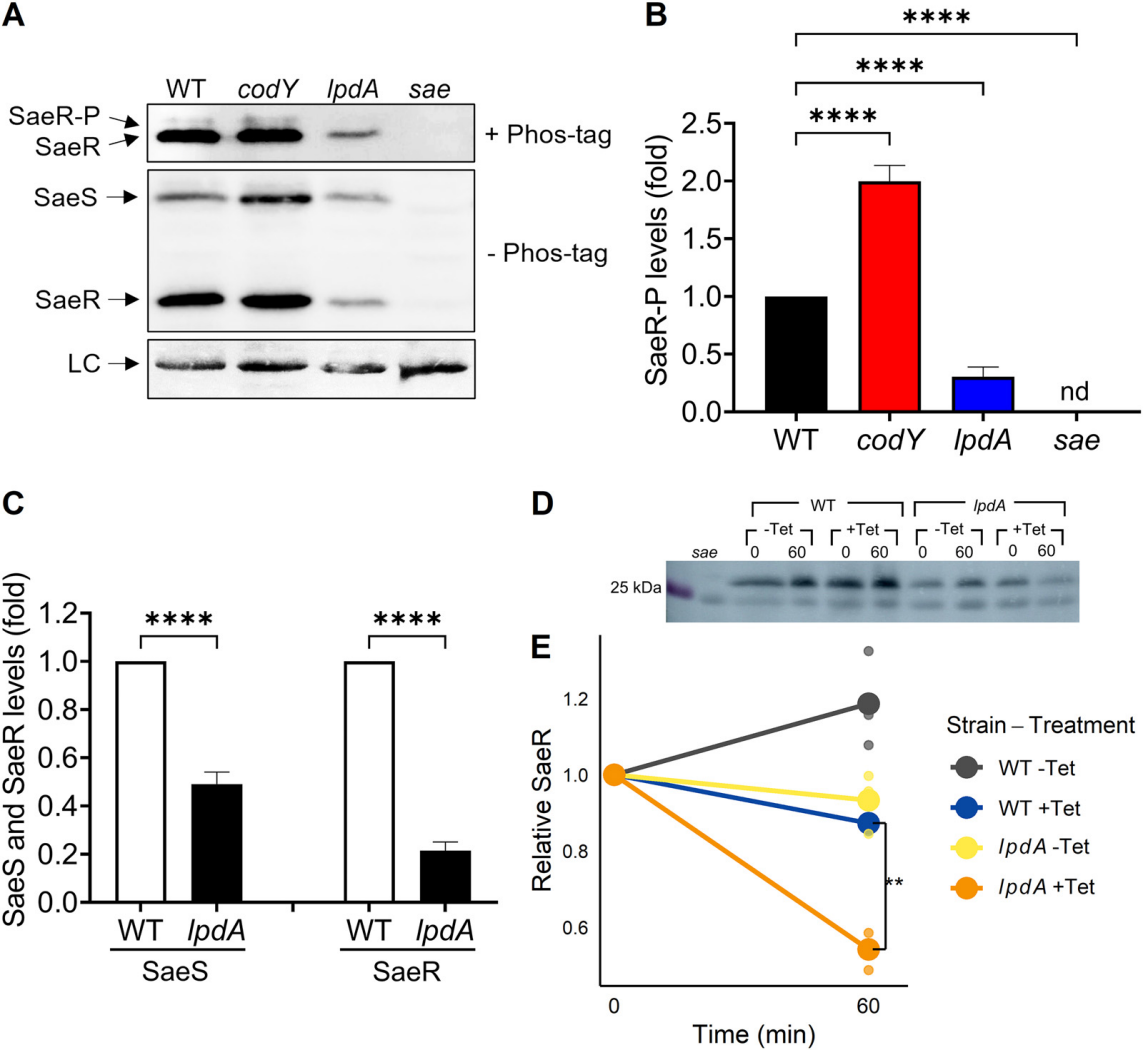
LpdA is required for promoting the state of phosphorylated SaeR *in vivo*. (A) Phos-tag Western blot (top) or Western blot (middle and bottom) analysis of crude extracts prepared from wild-type (WT), *codY*, *lpdA*, and *sae* USA300 strains grown in TSB medium for 5 h using antibodies recognizing SaeR. LC, loading control. (B) Levels of SaeR~P relative to SaeR~P from wild type. nd, not detected. (C) Levels of SaeS and SaeR from the *lpdA* mutant relative to those from the wild type were determined by SDS-PAGE and western blot analysis. All data are representative of three independent experiments, which produced similar results. One-way ANOVA with Tukey's post test was used for statistical analysis comparing SaeR~P levels. *n* > 3. ****, *P* < 0.0001. (D and E) Strains lacking the entire *sae* operon were complemented with *saeRS* under the control of the constitutive P3 promoter, in an otherwise wild-type (WT) and *lpdA* background; *sae* refers to the uncomplemented strain. Strains were grown for 4 h before supplementation with 15 μg mL^−1^ tetracycline; samples were removed for Western blotting at the beginning and end of a 1-h time course (0 and 60 min). Blots were normalized by total protein concentration. −Tet, no tetracycline; +Tet, tetracycline supplementation. (D) Representative blot of WT, *lpdA*, and *sae* whole-cell lysates. (E) Relative decrease in SaeR protein levels during 1-h translation blockade using tetracycline treatment. Lines connect the mean relative abundances at the beginning and end of the time course, and dots show individual biological replicates. *n* = 3. *, *P* < 0.05; **, *P* < 0.01, using ANOVA followed by Tukey’s HSD test.

**(iii) Disruption of *lpdA* reduces the amount of SaeS, SaeR, and SaeR~P *in vivo*.** We reasoned that the decrease in *sae*P1 promoter activity observed in the *lpdA* mutant might be due to reduced SaeR~P levels. To test this, we examined the amounts of SaeR~P in isogenic wild-type and *lpdA* S. aureus strains using Phos-tag gels. In agreement with the promoter activity data, the levels of SaeR~P were significantly reduced in the *lpdA* mutant (Fig. 7A and B). In addition, the total amounts of SaeS and SaeR in the *lpdA* mutant were also decreased (Fig. 7A and C) compared to those in the wild type. This could be due to reduced readthrough from saeP1, reduced stability of SaeR, or both. To measure SaeR stability in the *lpdA* mutant, we blocked translation using tetracycline and monitored protein levels at 4 h of growth and over a 1-h degradation time course. To compare protein stability levels without interference by the autoregulated P1 promoter, we complemented *sae* and *sae lpdA* strains with an integrated copy of *saeRS* under the control of the constitutive P3 promoter. A significantly higher percentage of SaeR was degraded over an hour of tetracycline treatment in the *lpdA* mutant, showing a clear stability defect (Fig. 7D and E). These results indicate that Sae activation requires LpdA to maintain the abundance of SaeS and SaeR, and SaeR is less stable in the *lpdA* mutant.

### BCFA deficiency alters expression of multiple TCSs and pathogenic potential.

**FIG 8:**
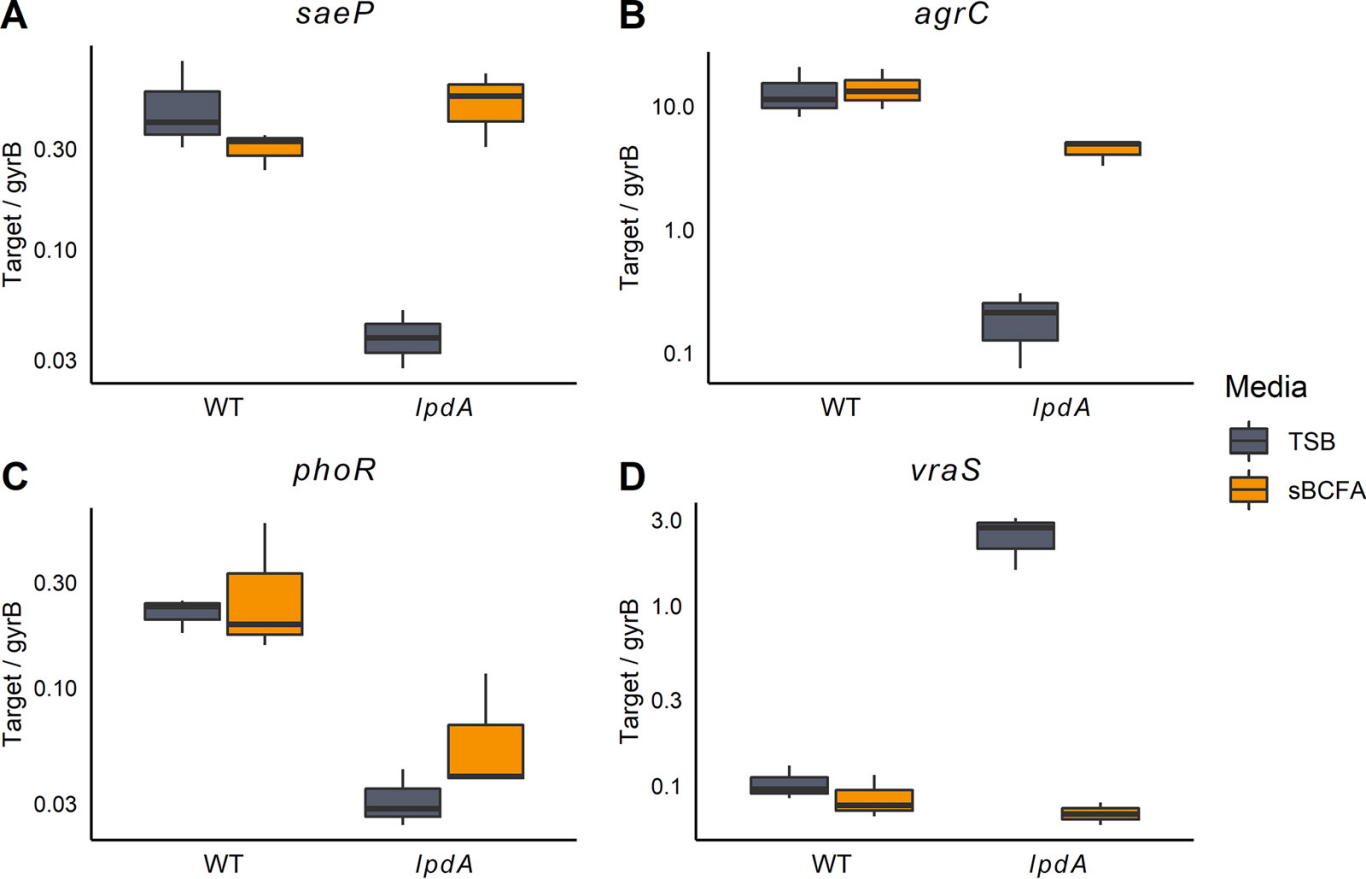
Disruption of *lpdA* significantly alters activity of four two-component systems. (A to D) RNA was extracted from strains grown in TSB with or without 0.5 mM sBCFAs for 5 h and used for RT-qPCR with primer sets specific to direct targets of 16 TCSs (4). Full results for all 16 systems are available in Fig. S4 in the supplemental material. Significantly different transcript abundance was observed between WT and *lpdA* strains in TSB medium for four systems, *saePQRS* (*saeP*), *agrCA* (*agrC*), *phoPR* (*phoR*), and *vraRS* (*vraS*) as determined by two-sample independent *t* tests with a Benjamini-Hochberg correction (α = 0.05, false-discovery rate = 0.05, *n* = 3). The *y* axis is log-scaled, and transcript abundances are normalized by the housekeeping gene gyrase B.

We next examined whether deficiencies in BCFA synthesis affect the activation and autoregulation of other TCSs (Fig. 8 and Fig. S4). Three TCSs had reduced activation in the *lpdA* mutant compared to the wild type: SaeRS, AgrCA, and PhoPR (Fig. 8A to C). AgrC responds to cell density and as such is likely less active in *lpdA* cultures because they reach lower cell densities than do wild-type cultures (15). That sBCFA supplementation only partially complements AgrC activation supports this hypothesis. PhoR responds to phosphate availability (51). Curiously, sBCFA supplementation does not rescue PhoR activation to wild-type levels (Fig. 8C). In contrast, VraS transcript number, required for oxacillin resistance (52), was increased >20-fold in the *lpdA* mutant compared to the wild type; sBCFA supplementation fully restored transcript levels to wild-type levels (Fig. 8D).

10.1128/mbio.01472-22.4 FIG S4 Impacts of *lpdA* disruption on transcription of all staphylococcal TCSs. RNA was extracted from strains grown in TSB with or without 0.5 mM sBCFAs for 5 h and used for RT-qPCR with primer sets specific to direct targets of 16 two-component systems. Significantly different transcript abundance normalized using *gyrB* was determined by two-sample independent *t* tests with a Benjamini-Hochberg correction (α = 0.05, false-discovery rate = 0.05, *n* = 3). Systems with significantly reduced sensor kinase activation in *lpdA* strain are boxed in blue, while the Vra TCS, with significantly higher sensor kinase activation in *lpdA* strain, is boxed in gold. *y* axis is log-scaled. Download FIG S4, TIF file, 0.4 MB. Copyright © 2022 Pendleton et al. 2022 Pendleton et al. https://creativecommons.org/licenses/by/4.0/ This content is distributed under the terms of the Creative Commons Attribution 4.0 International license.

**FIG 9:**
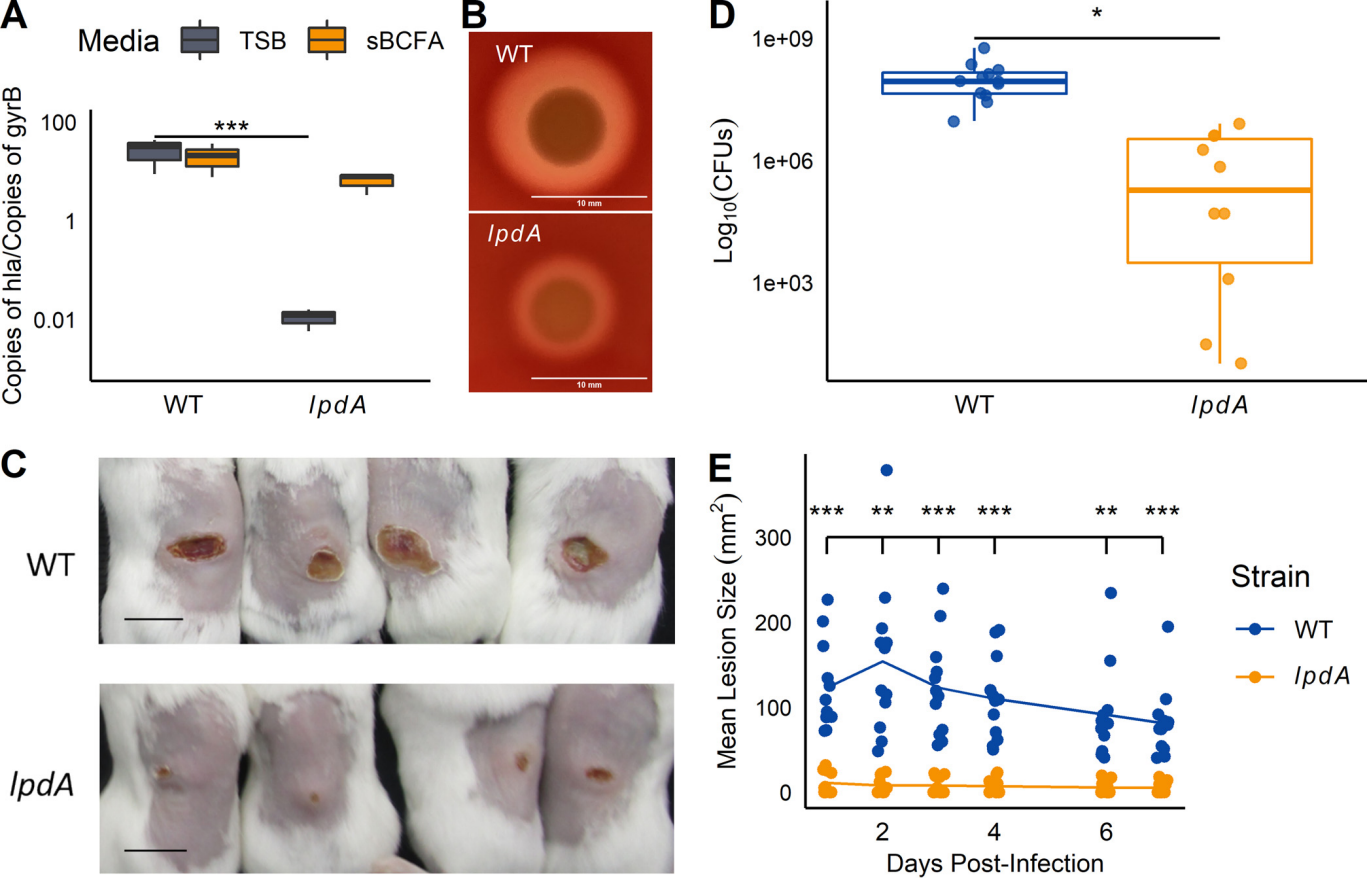
Hemolysin production and dermonecrosis are reduced in the *lpdA* mutant. (A) *hla* transcript levels were quantified in wild-type and *lpdA* cells grown for 5 h in TSB with or without 0.5 mM sBCFAs using RT-qPCR as described in Materials and Methods. Transcript levels are normalized by gyrase B (*gyrB*). *n* = 3. (B) *lpdA* mutants show reduced hemolysis on sheep blood agar plates. Representative image from 3 trials; bar = 10 mm. (C to E) Mice (*n* = 12) were infected subcutaneously with wild-type or *lpdA* S. aureus, and dermonecrotic lesions were measured for 7 days postinfection. (C) Images of representative skin lesions on day 7. Bar, 1 cm. (D) CFU were quantified 7 days after infection. (E) Mean lesion size over 7 days postinfection. *, *P* < 0.05; **, *P* < 0.01; ***, *P* < 0.001, ANOVA with Tukey *post hoc* test (A), Student’s *t* test (D), or ANOVA with repeated measures (E).

Finally, we wondered whether BCFA metabolism was important during infection. Previous work has shown that *hla* is a direct transcriptional target of SaeR (53). We reasoned that α-hemolysin production would be reduced in the *lpdA* mutant and that this would result in attenuated virulence. Indeed, using reverse transcription-quantitative PCR (RT-qPCR), we measured a nearly complete abrogation of *hla* transcript levels in the *lpdA* strain relative to the WT strain; the *lpdA* mutant also showed reduced hemolysis on blood agar plates (Fig. 9A and B). Further, infection with the *lpdA* mutant resulted in smaller dermonecrotic lesions and fewer CFU within dermal lesions (Fig. 9C to E). While Sae activity is clearly affected, we cannot exclude the possibility that other altered TCS activities or altered growth contributes to the virulence defect.

## DISCUSSION

Overexpressing the sensor kinase SaeS and response regulator SaeR alone does not alter the expression of target genes (21). Rather, the cellular fraction of the phosphorylated SaeR controls the production of virulence factors. As an intramembrane histidine kinase with a small extracellular component, SaeS likely responds to the overall conformation of its N-terminal transmembrane domains (14). As such, any molecule, endogenous or exogenous, can serve as a legitimate signal to upregulate virulence gene expression. This study aimed to understand how reductions in CodY activity might upregulate SaeRS activity. First, we established that CodY suppresses SaeS activity independent of binding to the P1 promoter and that the amount of SaeR~P in the *codY* mutant is increased, due to enhanced SaeS kinase activity. This increased SaeS activity is correlated with an increase in BCFAs in *codY* cell membranes; conversely, low BCFA levels in *lpdA* mutant cell membranes reduced SaeS activity, resulting in a huge decrease of SaeR and SaeR~P levels due at least in part to a decrease in SaeR stability. Knocking out *lpdA* prevented Sae activation in a *codY lpdA* mutant. The activity of other TCSs is also affected by low BCFA levels. We found that SaeS activation is dependent on BCFA synthesis specifically, independent of membrane fluidity or StrFAs. The BCFA-dependent defect in SaeS activation is also correlated with decreased toxin production and decreased virulence. These experiments uncover a novel link between CodY, BCFA synthesis, and TCS regulation. Experiments are ongoing to understand how CodY might upregulate Sae activity via BCFA synthesis; biosynthetic and salvaging pathways are likely involved. Since CodY also controls membrane fatty acid composition in B. subtilis, these linkages may occur across multiple Gram-positive bacteria.

Recent publications have uncovered new links between membrane composition and SaeS activation in S. aureus (49, 54). Previous work demonstrated that SaeS is inhibited by both saturated and unsaturated straight-chain fatty acids; in contrast, this study reveals BCFAs are activators of SaeS kinase activity *in vivo*. Interestingly, we found that SaeRS activity is restored in the *lpdA* mutant in late stationary phase, though cell density remains significantly lower (Fig. 5A and B). The synthesis of stationary-phase specific lipids and lipid-soluble pigments, including staphyloxanthin and cardiolipin, may account for this phase-specific rescue of SaeS activation (55, 56). Both cardiolipin and staphyloxanthin have been implicated in the structuring of protein-protein interactions and the formation of functional membrane microdomains (FMMs) (57, 58). As SaeS is similarly enriched in FMMs (58), experiments examining the contribution of these lipids to SaeR/S function are ongoing in our laboratory.

The full mechanism of BCFA-mediated control of Sae TCS activity remains unknown. We detected a significant decrease in SaeR and SaeR~P levels in the *lpdA* mutant, which can be explained in part due to reduced stability under BCFA-limited conditions. SaeS levels are also reduced (Fig. 7). It is conceivable that, as SaeS is a membrane protein, its stability may also be compromised, and SaeR instability may in fact be a consequence of SaeS degradation (17). At this time, our polyclonal antibodies against SaeS are not sufficiently specific to probe SaeS levels using translation blockade experiments; efforts to epitope tag SaeS are under way. We cannot rule out other possibilities. Lipids are emerging as important regulatory effectors in both bacterial and eukaryotic membrane receptors (59–61). The Escherichia coli chemotaxis receptor Tar is highly sensitive to changes to lipid composition, and membrane curvature determines its polar localization (60, 62). In E. coli, the osmolality receptor EnvZ is regulated by direct allosteric interactions with lipids through a glycine-rich motif (61). These interactions are critical for phosphotransfer from ATP (61). Our GC-FAME data suggest the primary driver of *lpdA-*dependent SaeS activation is anteiso-BCFAs, though isobutyric acid supplementation can also rescue Sae activity (Fig. 5F and data not shown). It is possible that anteiso-BCFAs influence the ATP-binding domain of SaeS to increase ATP turnover or convert SaeS from a phosphatase-dominant to a kinase-dominant state. Liposome reconstitution using SaeS and full-length BCFAs as well as purification of epitope-tagged SaeS from native membranes followed by mass spectrometry could help determine which specific BCFAs are interacting with SaeS and a biochemical explanation for the effect (63, 64). Interacting proteins that might bind SaeS and BCFAs or otherwise facilitate SaeS interaction with BCFAs could also be identified using this approach. Phosphorylation and stability of SaeR may also be controlled by BCFA-responsive cytoplasmic factors. Indeed, the biochemical mechanisms of how branched-chain fatty acids modulate Sae activity are an active area of research in our laboratory.

## MATERIALS AND METHODS

### Bacterial strains and growth conditions.

S. aureus strains used in this study are listed in Table S2 in the supplemental material. Unless otherwise noted, S. aureus strains were cultured in tryptic soy broth (TSB) containing 0.25% (wt/vol) dextrose (BD Biosciences), and Escherichia coli strains were grown in modified Lennox (L) medium containing 10 g L^−1^ tryptone, 5 g L^−1^ yeast extract, and 5 g L^−1^ sodium chloride. B. subtilis strains were grown in Lenox broth (Difco) with 14 mM glucose, and L. monocytogenes strains were grown in brain heart infusion medium (Difco). C. difficile strains were grown in brain heart infusion medium with 5 g L^−1^ yeast extract and 0.1% l-cysteine in a Coy anaerobic chamber containing 5% CO_2_, 10% H_2_, and 85% N_2_. Unless otherwise noted, all strains were grown at 37°C. When necessary, media were solidified with agar (1.5% [wt/vol]) and supplemented with the following antibiotics at the indicated concentrations: ampicillin (Ap), 50 μg mL^−1^; chloramphenicol (Cm), 5 to 10 μg mL^−1^; erythromycin (Erm), 5 μg mL^−1^; tetracycline (Tc), 1.5 μg mL^−1^; or kanamycin (Km), 100 μg mL^−1^ for S. aureus or 50 μg mL^−1^ for E. coli. When indicated, media were supplemented with short, branched-chain fatty acids (sBCFAs; 2-methylbutyric acid, isovaleric acid, and isobutyric acid) to a final concentration of 0.5 mM. Experiments requiring exponential growth were performed in 125-mL DeLong shake flasks with 12.5 mL TSB (10:1 flask/medium ratio). All experiments used a double-back dilution scheme to ensure steady-state growth. Briefly, precultures in 125-mL flasks were inoculated from overnight cultures to an optical density at 600 nm (OD_600_) of 0.05 and incubated in a gyratory water bath shaking at 240 rpm. Cultures were grown to exponential phase to an OD_600_ of ~1.0 and diluted into fresh TSB to an OD_600_ of 0.05. Exponential-phase samples (for *nuc-gfp* reporter assays, *codY* membrane vesicle preparation, and GC-FAME sample collection) were collected at an OD_600_ of 0.5 ± 0.05. For green fluorescent protein (GFP) reporter assays in the *lpdA* mutant, membrane vesicle preparation, reverse transcription-quantitative PCR (RT-qPCR), and Western blotting, cells were grown for 5 h after their second dilution in TSB. Cell growth was monitored by measuring OD_600_ using an Amersham Ultraspec 2100 Pro UV-visible spectrophotometer. Unless otherwise noted, chemical reagents were purchased from Millipore-Sigma.

10.1128/mbio.01472-22.7 TABLE S2 Strains used in this study. ^a^Unless noted otherwise, strains were constructed during the course of this study. Download Table S2, DOCX file, 0.03 MB. Copyright © 2022 Pendleton et al. 2022 Pendleton et al. https://creativecommons.org/licenses/by/4.0/ This content is distributed under the terms of the Creative Commons Attribution 4.0 International license.

### Recombinant DNA and genetic techniques.

Oligonucleotides for this study were synthesized by Integrated DNA Technologies (Coralville, IA) and are listed in Table S3. Restriction enzymes, T4 DNA ligase, *Taq* ligase, Q5 DNA polymerase, and T5 exonuclease, as well as PCR cleanup and gel extraction kits, were purchased from New England Biolabs (NEB). Plasmid and genomic DNA (gDNA) miniprep kits were purchased from Promega. Complementation plasmids were constructed using Gibson assembly as described previously (65). Plasmids used in this study are listed in Table S4. E. coli NEB 5α (NEB) or E. coli Mix and Go DH5α (Zymo) was used as a host for plasmid constructions, and all plasmids were confirmed by PCR or restriction digestion in addition to Sanger sequencing (Genewiz/Azenta). Plasmids from E. coli were introduced into S. aureus strain RN4220 by electroporation (66) prior to transfer into clinical isolates or directly into clinical isolates after passaging plasmids through E. coli strain IM08B (67). As needed, plasmids and mutant alleles conferring antibiotic resistance phenotypes were moved between S. aureus strains via ϕ11-mediated transduction or ϕ85-mediated transduction (68).

10.1128/mbio.01472-22.8 TABLE S3 Oligonucleotides used in this study. Unless noted otherwise, oligonucleotides were designed during the course of this study. Download Table S3, DOCX file, 0.02 MB. Copyright © 2022 Pendleton et al. 2022 Pendleton et al. https://creativecommons.org/licenses/by/4.0/ This content is distributed under the terms of the Creative Commons Attribution 4.0 International license.

10.1128/mbio.01472-22.9 TABLE S4 Plasmids used in this study. Unless noted otherwise, plasmids were constructed during the course of this study. Download Table S4, DOCX file, 0.02 MB. Copyright © 2022 Pendleton et al. 2022 Pendleton et al. https://creativecommons.org/licenses/by/4.0/ This content is distributed under the terms of the Creative Commons Attribution 4.0 International license.

### Plasmid constructions.

For plasmid pAP3 (BKDH gene cluster), the coding sequence for *bkdA1*, *bkdA2*, and *bkdB* (3,318 bp) was amplified from LAC gDNA using primers oAP31 and oAP32 with Q5 DNA polymerase. The pAP1 plasmid, including the *sarA* P1 promoter and *lpdA* open reading frame (ORF) (6,294 bp), was amplified and linearized using primers oAP33 and oAP34. These primers were designed to add 25-bp overlapping regions for later Gibson assembly. PCR products were treated with DpnI (NEB) for 1 hour at 37°C. The amplified PCR products were then subjected to Gibson assembly and inserted into E. coli DH5α.

### Nuclease activity quantification via FRET.

Secreted nuclease activity was quantified using a fluorescence resonance energy transfer (FRET) assay described previously (69). In brief, cells were grown to exponential phase and centrifuged. Culture supernatants (0.7 mL) were sterilized using 0.22-μm Spin-X centrifuge tube filters (Corning) and frozen at −20°C. The single-stranded oligonucleotide FRET substrate was diluted to a concentration of 2 μM in buffer A (20 mM Tris [pH 8.0], 0.5 M CaCl_2_). The substrate was mixed 1:1 with the nuclease-containing supernatants. Fluorescence, indicative of the substrate cleavage by nuclease, was measured at 30°C using a computer-controlled Tecan Infinite F200 Pro instrument equipped with 535-nm excitation and 590-nm emission filters. The relative fluorescence units were converted to units of nuclease activity by interpolation using a standard curve generated with purified micrococcal nuclease enzyme (Worthington Biochemicals). Typically, sterilized culture supernatants were diluted 1:10 in water and then serially diluted 2-fold in water up to 14 times to ensure they lay within the linear regression of the standard curve samples.

### RNA extraction.

RNA extraction was performed as previously described (9). Briefly, cells were grown as described above to exponential phase (OD_600_ of 0.5), at which time a 4-mL sample was quenched with an equal volume of a 1:1 (vol/vol) mixture of ethanol-acetone prechilled to −20°C. The quenched samples were immediately frozen on dry ice and then transferred for long-term storage to a −80°C freezer. Once thawed, cells were pelleted, washed twice with TE buffer (10 mM Tris-Cl [pH 8], 1 mM EDTA), resuspended in TRIzol (Thermo Fisher Scientific), and then mechanically disrupted using a Precellys 24 homogenizer (Bertin Technologies) with three 30-s pulses at 6,800 rpm. Samples were incubated for 1 min on wet ice between pulses. Nucleic acids from each sample were extracted using a Direct-Zol kit (Zymo Research), and any contaminating genomic DNA was digested in each sample using a Turbo DNA-free DNase removal kit (Ambion), according to the manufacturer’s instructions.

### RT-qPCR.

Reverse transcription followed by quantitative PCR was performed essentially as previously described (9). The SensiFAST cDNA synthesis kit (Thomas Scientific) and random hexamer primers were used to reverse transcribe 250 ng of total RNA from each sample, as per the manufacturer’s instructions. Reactions were performed in parallel without reverse transcriptase (−RT) to control for residual DNA contamination during the quantitative PCR. The abundance of specific cDNAs in each sample, reflecting the original number of specific transcripts, was determined using a C1000 thermal cycler fitted with a CFX96 detection module, SsoAdvanced Universal SYBR green Supermix (Bio-Rad), and target-specific oligonucleotides at a concentration of 400 nM each. Reaction mixtures were incubated at 98°C for 2 min and then cycled between 98°C and 60°C. No-template and −RT reactions were run on each plate as controls. Threshold fluorescence measurements were converted to transcript abundance using standard curves generated from serial dilutions of genomic DNA spanning at least 5 orders of magnitude. Target transcript abundance was normalized to *rpoC* or *gyrB* loading controls, as the abundance of this transcript remains constant (<2-fold change) across all strains and conditions analyzed.

### Analysis of fatty acid composition of S. aureus strains.

S. aureus strains were grown to exponential phase; at an OD_600_ of 0.5, 10 mL of each culture was pelleted, washed twice with phosphate-buffered saline (PBS), and stored at −80°C. Fatty acids were saponified and methylated and then subjected to gas chromatographic (GC) analysis of fatty acid methyl esters (FAMEs) on a fee-for-service basis by Microbial ID (Newark, DE) or the Center for Microbial Identification and Taxonomy (Norman, OK).

### Measurement of membrane fluidity.

Membrane fluidity was measured using membrane polarization as previously described (70). Briefly, cultures were grown exponentially to an OD_600_ of 0.5, at which point bacteria were pelleted by centrifugation at 25°C and washed twice with phosphate-buffered saline (PBS), pH 7.4. Cells were resuspended in PBS containing 2 μM 1,6-diphenyl-1,3,5-hexatriene (DPH; Sigma-Aldrich) to an OD_600_ of 0.4. A 2 mM DPH stock solution was made in tetrahydrofuran before diluting into PBS. Samples were incubated at 37°C for 1 h. Fluorescence polarization was then measured in quartz cuvettes using a PerkinElmer LS55 spectrofluorometer with excitation and emission wavelengths of 360 and 426 nm, respectively, and blanked using bacterial cells processed and resuspended in PBS in the same way with equivalent amounts of tetrahydrofuran but without DPH. Samples were excited using vertically polarized light, and the emitted light was detected through polarized filters. Polarization was calculated as previously described (70, 71).

### GFP reporter assays.

Cells carrying the indicated reporter fusions were grown from an OD_600_ of 0.05 to specified times after their second dilution in TSB with or without supplements: 0.5 mM BCFAs, 0.1% benzyl alcohol (BnOH), 5 μg mL^−1^ human neutrophil peptide 1 (HNP-1), 10 mg mL^−1^ fatty-acid-free bovine serum albumin (BSA), or 0.5 mM 15:0 anteiso-fatty acid (Avanti). For complementation, S. aureus COL, and fluidizing condition experiments, cells were grown with the 10:1 flask/medium ratio in DeLong flasks. For HNP activation and full-length 15:0 anteiso-BCFA experiments, cells were grown in 1 mL medium in 16- by 125-mm sterile borosilicate glass test tubes. For HNP activation, cells were grown for 2 h in DeLong flasks before being split into test tubes and activated with HNP-1 or carrier (15 mM Tris-Cl, pH 8) and grown an additional 2.5 h before GFP fluorescence was measured. Cell culture samples (400 to 800 μL) were pelleted by centrifugation, washed twice with phosphate-buffered saline (pH 7.4), and resuspended in 400 μL of phosphate-buffered saline (pH 7.4) (because *lpdA* strains reach lower OD values at 5 h, larger volumes of cells would be pelleted to increase OD when measuring in the well plate). Fluorescence was measured using a computer-controlled BioTek Synergy H1 plate reader exciting samples at 485 nm and measuring emission at 535 nm. Relative fluorescent units (RFUs; GFP fluorescence/OD_600_) were calculated by subtracting the fluorescence of plain phosphate-buffered saline and dividing by OD_600_ to correct for cell density.

### Isolation of membrane vesicles.

Membrane vesicles were prepared as described previously with a slight modification (20). Wild-type and *codY* mutant cells of USA300 LAC or UAMS-1 were grown for 5 h in TSB. Cell pellets were frozen and resuspended in TSM (50 mM Tris [pH 8.0], 0.5 M sucrose, 10 mM MgCl_2_) containing 40 μg mL^−1^ lysostaphin and incubated for 20 min at 37°C. An additional 2 mL of membrane lysis buffer (100 mM Tris [pH 8.0], 100 mM NaCl, 10 mM MgCl_2_) was added, and cells were lysed by sonication (Fisherbrand model 120 sonic dismembrator). A 100-μL sample of whole-cell lysate was saved for Western blot analysis, and the remaining lysate including the membrane fraction was centrifuged at 7,000 × *g* for 5 min. The membrane fraction was recovered via ultracentrifugation at 82,700 × *g* for 30 min (Optima XPN-90 ultracentrifuge; Beckman). The pellet was washed and pelleted through three additional rounds of ultracentrifugation (82,700 × *g*, 30 min each round), first in 20 mM Tris (pH 8.0) plus 2 M KCl, then in 20 mM Tris (pH 8.0) plus 5 mM EDTA, and then in 20 mM Tris (pH 8.0). Finally, the pellet was resuspended in TKMG buffer (50 mM Tris [pH 8.0], 50 mM KCl, 1 mM MgCl_2_, 25% [vol/vol] glycerol) and stored at −80°C until use.

### SaeS and SaeR purification.

The SaeR-His_6_ protein was overproduced in E. coli BL21(DE3) harboring pET22b-*saeR*. Overnight cultures were inoculated into fresh LB broth, and SaeR-His_6_ was overexpressed by the addition of 1 mM isopropyl-1-thio-β-d-galactopyranoside (IPTG) to the fresh culture. The bacterial culture was further incubated at 37°C for 6 h. SaeR-His_6_ was purified with nickel-nitrilotriacetic acid (Ni-NTA) column chromatography (Qiagen) by following the manufacturer’s recommendations. The purified SaeR-His_6_ protein was exchanged with TBS buffer (10 mM Tris-HCl [pH 7.5], 138 mM NaCl, 2.7 mM KCl), followed by TBS buffer containing 25% (vol/vol) glycerol, and concentrated using an Amicon Ultra-15 centrifugal filter unit (molecular weight [MW] cutoff of 10,000; Millipore). The MBP-SaeS protein was purified as described before (20). Protein concentration was determined by the bicinchoninic acid (BCA) assay (Bio-Rad), and the purified proteins were stored at −80°C until used.

### Kinase activity assays.

Three to five hundred micrograms of membrane vesicles was mixed with 10 μM SaeR-His_6_ protein in TKM buffer (50 mM Tris [pH 8.0], 50 mM KCl, 1 mM MgCl_2_) at room temperature for 5 min. The reaction was started by incubating with 0.1 mM ATP containing 20 μCi of [γ-^32^P]ATP (3,000 Ci mmol^−1^; PerkinElmer) at 37°C as described before (72). A 9-μL aliquot was mixed with 6× SDS sample buffer at different time points to stop the reaction. The phosphorylated SaeR-His_6_ proteins were separated by 12% Bis-Tris SDS-PAGE and determined by quantifying the ^32^P-labeled species using an Amersham Typhoon biomolecular imager and a phosphorimaging plate (Fujifilm) followed by quantification with Multi Gauge software V 3.0 (Fujifilm). The data were fitted using nonlinear regression to a one-phase exponential association (Prism ver. 9; GraphPad). All data correspond to the mean ± standard error of the mean (SEM) from three independent experiments.

### Stability assays.

After double-back dilution, strains were grown for 4 h, at which point they were supplemented with 15 μg mL^−1^ tetracycline (a nonsupplemented control was included). Samples were immediately removed, pelleted at 2°C, and frozen in liquid nitrogen (*T* = 0). After 1 h of additional incubation, samples were again pelleted and frozen for Western blotting.

### Western blotting.

Protein concentrations of whole-cell lysates and membrane fractions were measured using the BCA assay. Cells were resuspended in 20 mM Tris-HCl buffer (pH 8.0) and lysed with lysostaphin (40 μg mL^−1^) in a 37°C heat block for 30 min. SDS loading buffer at 5× was added to the cell lysates, followed by heating for 5 min. Samples were subjected to 12% SDS-PAGE, and proteins were transferred to polyvinylidene difluoride PVDF membranes (Cytiva). Membranes were blocked in 5% (wt/vol) skim milk in TBST (20 mM Tris-HCl, 150 mM NaCl, and 0.05% [vol/vol] Tween 20, pH 7.6) for 1 h. Membranes were washed three times with TBST and incubated with rabbit polyclonal antibodies specific to SaeR (1:1,000 dilution) and SaeS (1:1,000 dilution) in blocking buffer for 1 h (20). Membranes were washed with TBST and incubated with StarBright Blue 700 goat anti-rabbit IgG (1:3,500; Bio-Rad) for 1 h. After a brief wash in TBST, the signals were visualized using an Amersham ImageQuant 800. The densities (mean intensity per unit area) of the SaeS and SaeR protein bands were determined by quantification with Multi Gauge software (Fujifilm).

### Detection of phosphorylated SaeR *in vivo* and *in vitro*.

SaeR and SaeR~P were separated on 12% polyacrylamide gels containing acrylamide–Phos-tag ligand (Wako Laboratory Chemicals) as described by the manufacturer. Gels were copolymerized with 50 μM Phos-tag acrylamide and 100 μM MnCl_2_. Whole-cell extracts were prepared as described previously (38) and normalized by an optical density at 600 nm. The samples were electrophoresed on Phos-tag gels with standard running buffer (0.1% [wt/vol] SDS, 25 mM Tris, 192 mM glycine) at 4°C under constant voltage (150 V) for 2 h. The gels were washed twice for 15 min with transfer buffer (25 mM Tris [pH 8.3], 192 mM glycine, 20% methanol) containing 1 mM EDTA and once with transfer buffer without EDTA to remove EDTA. Proteins were then transferred to PVDF membranes (0.45 mm; Cytiva) and analyzed by immunoblotting using polyclonal rabbit antibodies to SaeR (1:1,000) and SaeS (1:1,000). Membranes were washed three times with TBST and incubated with StarBright Blue 700 goat anti-rabbit IgG (1:3,500; Bio-Rad) for 1 h. After a brief wash in TBST, the signals were visualized using an Amersham ImageQuant800. The densities of the SaeR~P protein bands were determined by quantification with Multi Gauge software (Fujifilm). The data are representative of at least three independent experiments, which produced similar results.

To generate phosphorylated SaeR *in vitro*, 5 μM MBP-SaeS was incubated with 10 μM SaeR-His_6_ in TKM buffer in the absence (a negative control) or presence of 1 mM ATP at 37°C for 1 h. The reaction was stopped by the addition of 5× SDS loading buffer. Phosphorylated and unphosphorylated forms of SaeR were separated on 12% Phos-tag gels and visualized by Coomassie blue staining.

### Skin and soft tissue infection models.

Animal experiments were approved by the Animal Care and Use Committee at the Abigail Wexner Research Institute at Nationwide Children’s Hospital. The mouse model of S. aureus SSTI has been reported previously (34, 35) Female BALB/c mice were purchased from Taconic Biosciences and infected at 7 to 8 weeks of age. Hair was removed using depilatory cream (Nair) 1 day prior to infection. Bacteria were revived from frozen stocks, incubated at 37°C overnight, and subcultured into tryptic soy broth and grown at 37°C overnight with shaking (250 rpm). On the morning of infection, the overnight culture was diluted in fresh TSB and grown to an OD_600_ of 1.8 (approximately 3 h), following which the bacteria were centrifuged and washed, and the pellet was resuspended in phosphate-buffered saline at a concentration of 1.5 × 10^7^ CFU in 50 μL. For inoculation, mice were sedated with intraperitoneal ketamine and xylazine. Inoculation was performed by subcutaneous injection of 50 μL of S. aureus. Following inoculation, mice were allowed to awaken and had free access to food and water. Lesion sizes were quantified daily for 7 days by digital photography. Mice were euthanized by forced CO_2_ inhalation on day 7, and skin lesions were excised for bacterial quantification. Lesions were homogenized, and serial dilutions of the homogenate were plated on mannitol salt agar and incubated at 37°C for 24 h prior to colony counts.

### Statistical methods.

Statistical significance was calculated using measurements from at least three biological replicates, which were started from separate colonies and grown as separate cultures. Technical replicates were averaged, and statistical tests were run comparing only biological replicates. When comparing between-group averages, one-way and two-way analysis of variance (ANOVA) was used; for time course experiments, two-way ANOVA with repeated measures was used to assess differences. ANOVAs, if significant, were followed by Tukey’s honestly significant difference (Tukey’s HSD) tests for pairwise comparisons. When comparing only wild type and *lpdA* mutant under a single condition, Student’s *t* tests were used with either Bonferroni or Benjamini-Hochberg corrections. Statistical significance was assumed at a *P* value of <0.05. All statistical analyses were run either in R (version 4.0.2; RStudio version 1.3.959) using base functions (73), the tidyverse packages (74), and the rstatix package (75) or in Prism ver. 9 (GraphPad).
