# Supplementary figures and images for: Regulation of the Sae Two-Component System by Branched-Chain Fatty Acids in Staphylococcus aureus
Source: mBio. 2022 Sep 22;13(5):e01472-22. doi: 10.1128/mbio.01472-22 (PMC9600363; doi:10.1128/mbio.01472-22)

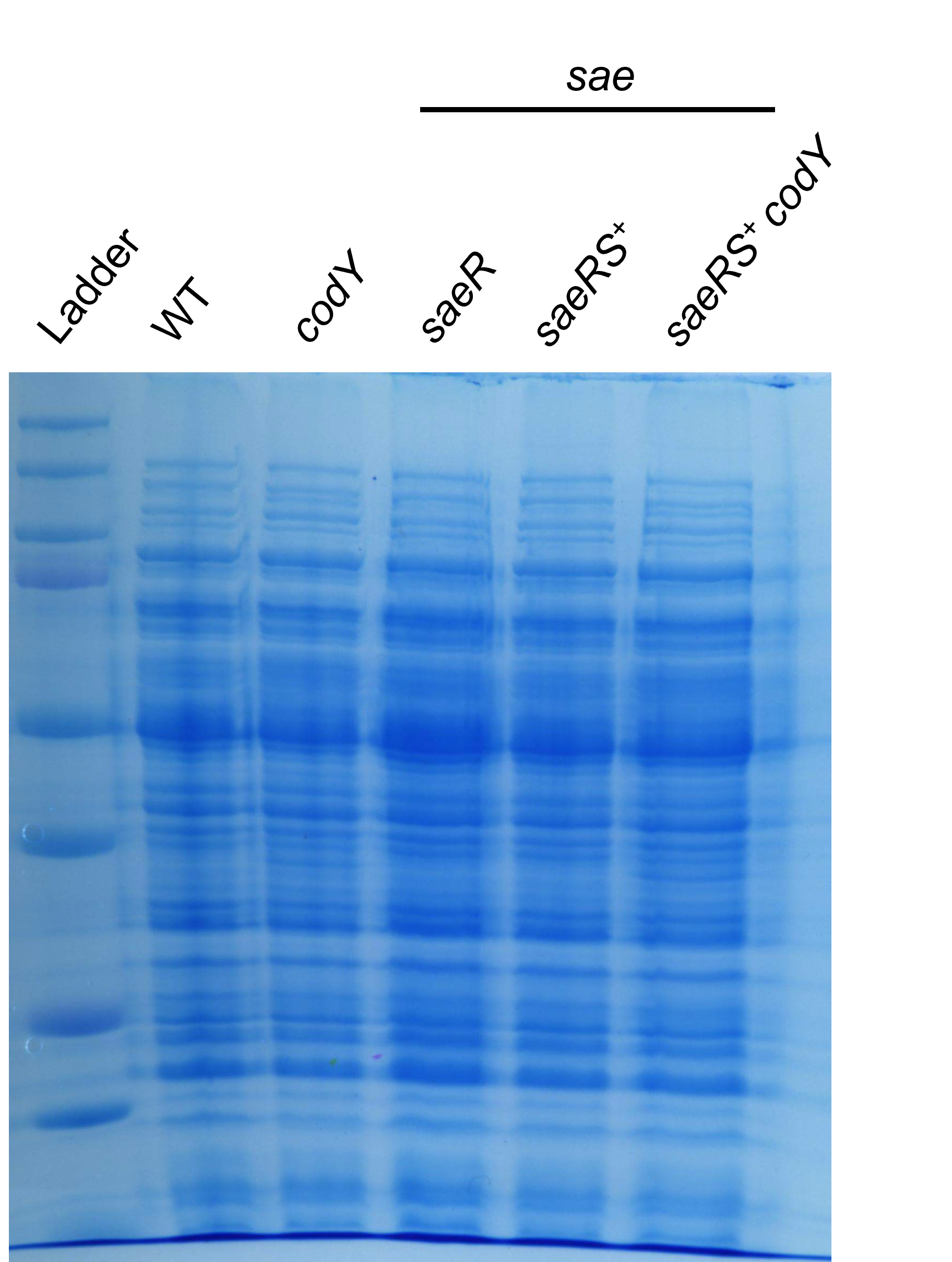

Supplement: FIG S5 [file mbio.01472-22-s0005.tif]

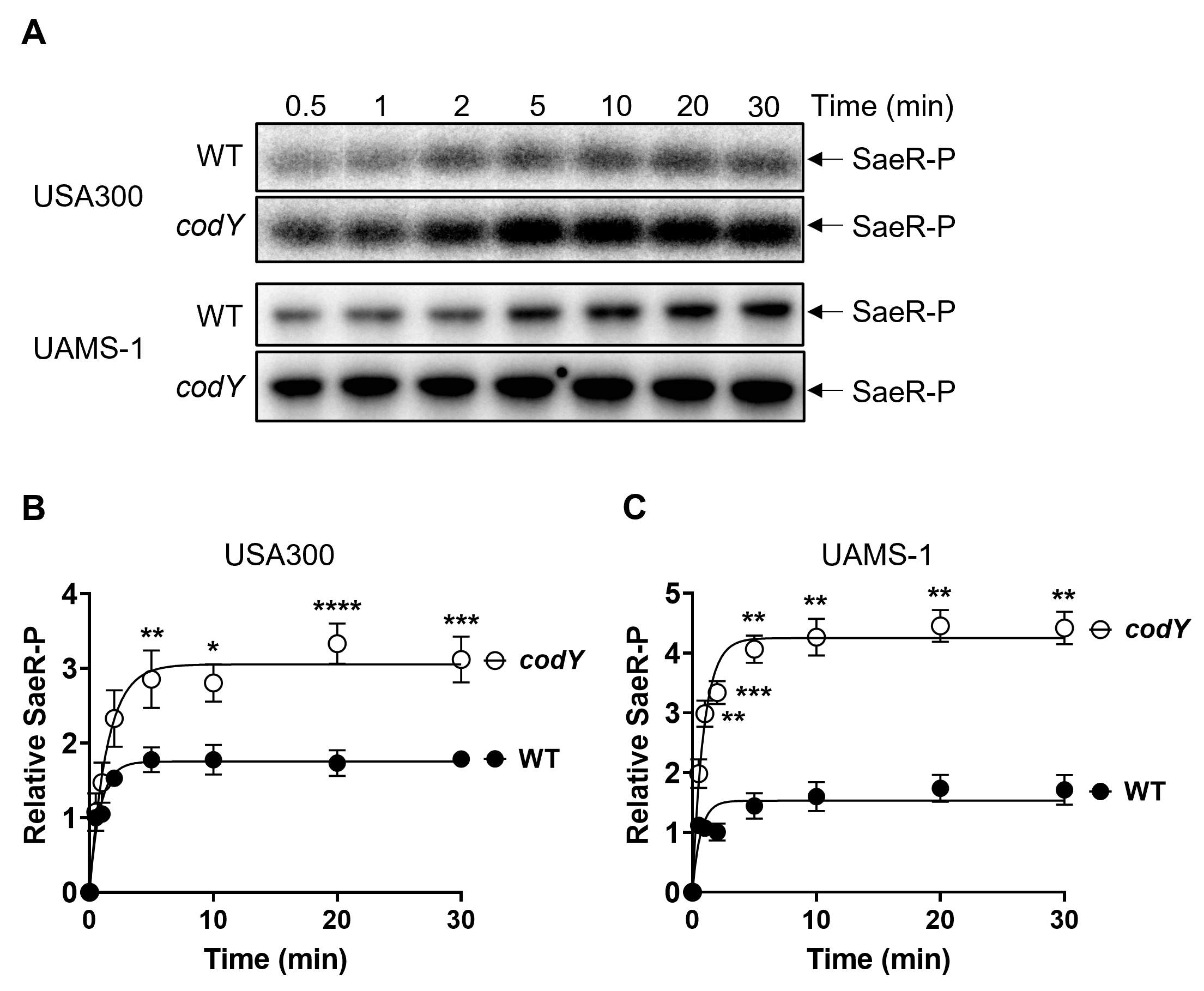

Supplement: FIG S1 [file mbio.01472-22-s0001.tif]

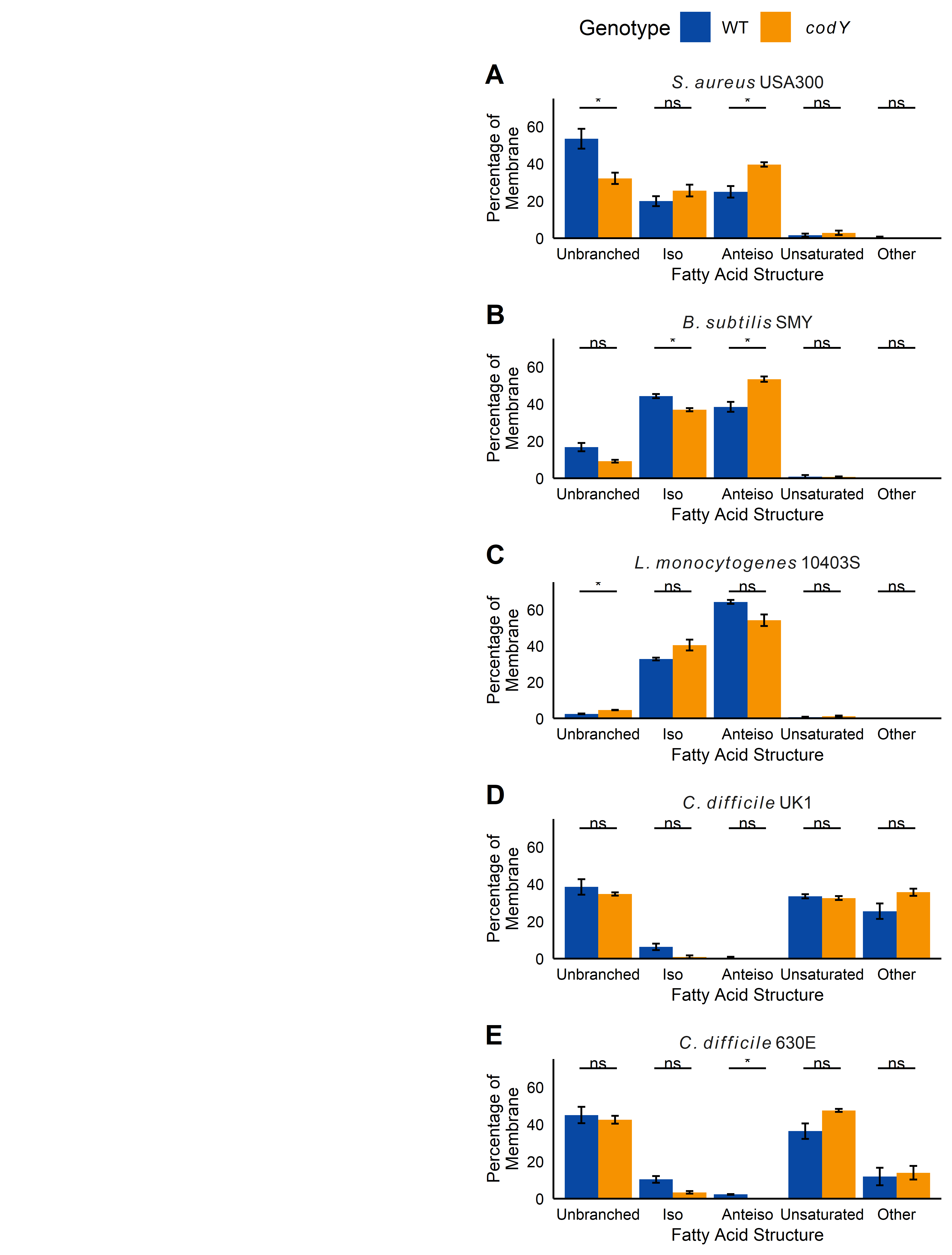

Supplement: FIG S2 [file mbio.01472-22-s0002.tif]

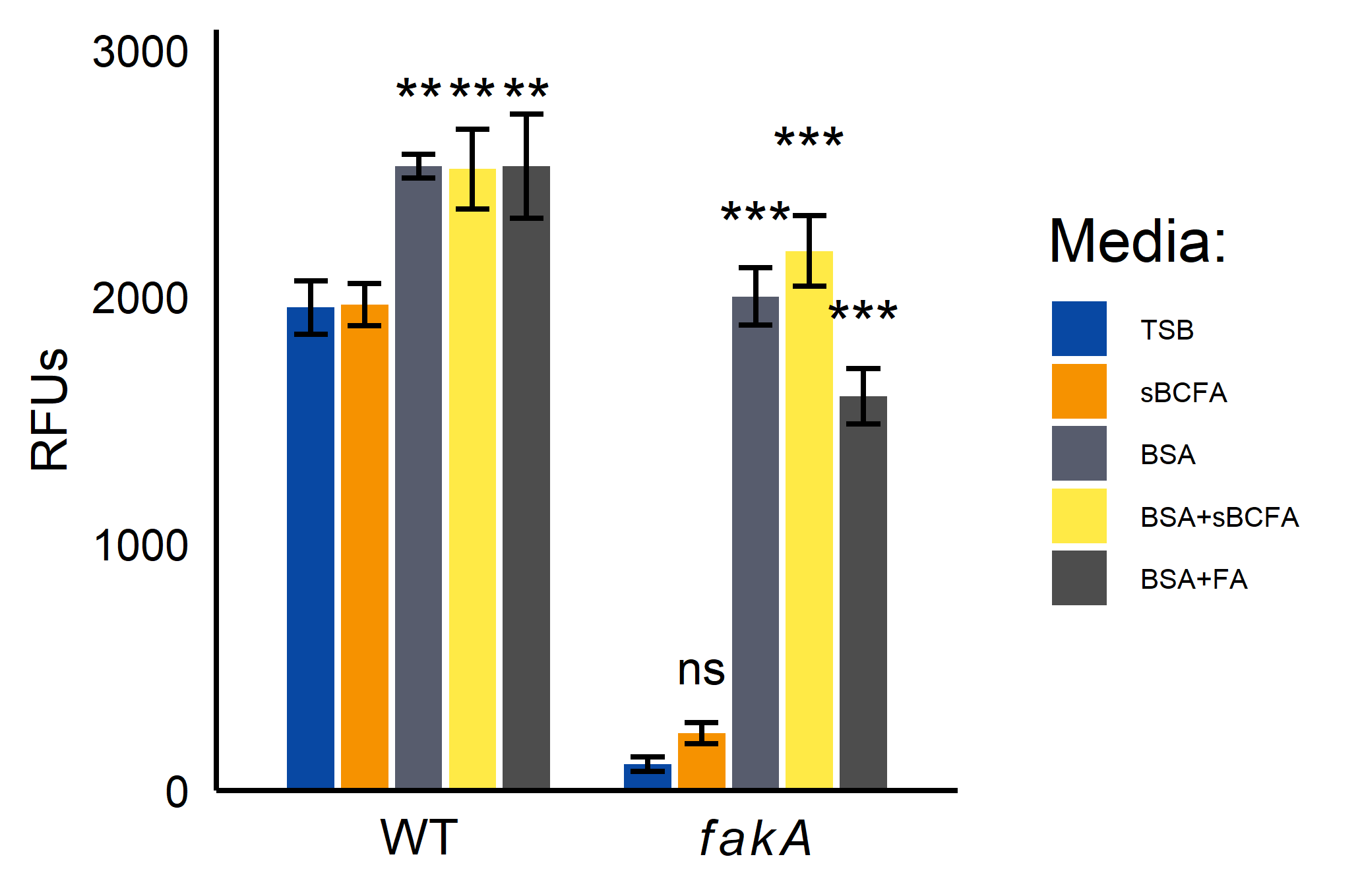

Supplement: FIG S3 [file mbio.01472-22-s0003.tif]

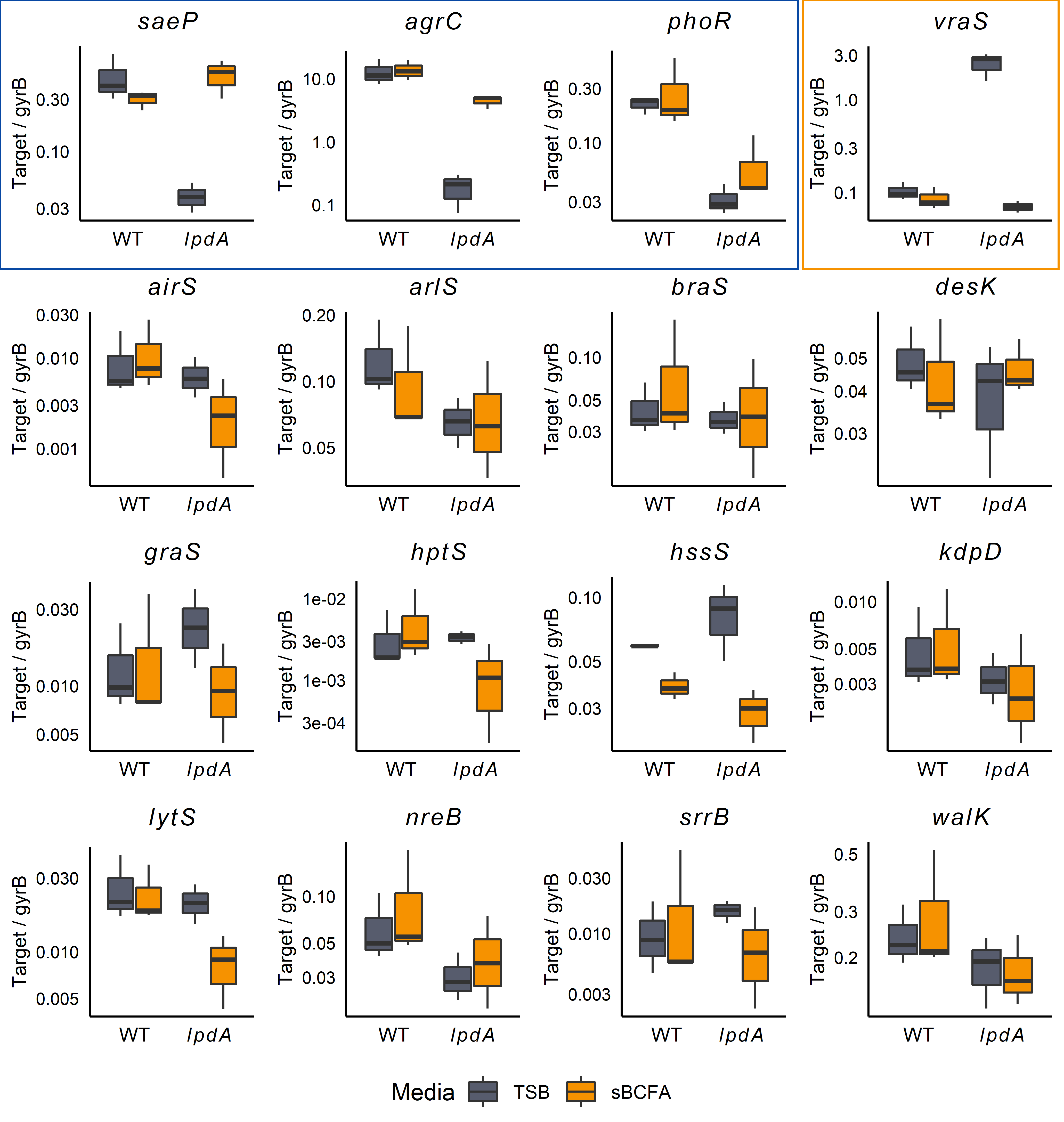

Supplement: FIG S4 [file mbio.01472-22-s0004.tif]
