# Supplementary material for: Regulation of the Sae Two-Component System by Branched-Chain Fatty Acids in Staphylococcus aureus
Source: mBio. 2022 Sep 22;13(5):e01472-22. doi: 10.1128/mbio.01472-22 (PMC9600363; doi:10.1128/mbio.01472-22)
Supplement: TABLE S1 [file mbio.01472-22-s0006.docx]

| Fatty Acid | Percentage Difference ± SD |
| --- | --- |
| 14:0 iso | 3.71 ± 1.04 |
| 15:0 iso | -5.26 ± 0.41 |
| 16:0 iso | 10.67 ± 1.83 |
| 17:0 iso | -1.45 ± 0.16 |
