# Supplementary material for: Regulation of the Sae Two-Component System by Branched-Chain Fatty Acids in Staphylococcus aureus
Source: mBio. 2022 Sep 22;13(5):e01472-22. doi: 10.1128/mbio.01472-22 (PMC9600363; doi:10.1128/mbio.01472-22)
Supplement: TABLE S2 [file mbio.01472-22-s0007.docx]

| **Strain** | **Genotype or description** | **Source or reference*^a^*** |
| --- | --- | --- |
| NE1896 | USA300 JE2 *lpdA*::ɸNΣ | (1) |
| NE1354 | USA300 JE2 *hla::*ɸNΣ | (1) |
| NE229 | USA300 JE2 *fakA(vrfB*)::ɸNΣ | (1) |
| RN4220 | Restriction-deficient, highly transformable | (2) |
| SRB337 | UAMS-1 | (3) |
| SRB372 | UAMS-1 *codY*::φNΣ | (4) |
| SRB687 | USA300 LAC CA-MRSA EmS (AH1263) ("LAC") | A.R. Horswill |
| SRB688 | *nuc*::*ltrB* | (5) |
| SRB746 | LAC *codY*::φNΣ | (4) |
| SRB761 | LAC *saeR*::φNΣ [*erm::tet*(M)] *codY*::φNΣ | (4) |
| SRB783 | LAC *saeR*::φNΣ [*erm::tet*(M)] |  |
| SRB892 | LAC *codY*::φNΣ [*erm::tet*(M)] | (6) |
| SRB986 | LAC HOU0087:: *nuc-gfp* φ11*att*::pLL29 *sarA*p1-tdTomato | (7) |
| SRB987 | LAC *codY*::φNΣ HOU0087:: *nuc-gfp* φ11*att*::pLL29 *sarA*p1-tdTomato | (7) |
| SRB1176 | LAC pKM11 | (8) |
| SRB1395 | LAC Δ*sae* FL54a::pCL55 P*sae*P3-*saeRS* | (9) |
| SRB1411 | LAC Δ*sae* FL54a::pCL55 *Psae*P3*-saeRS* HOU0087:: *nuc*-gfp | (9) |
| SRB1476 | *Bacillus subtilis* SMY | P. Schaeffer via AL Sonenshein |
| SRB1477 | *B. subtilis* SMY *codY::spc* | (10) |
| SRB1478 | *Listeria monocytogenes* 10403s | D. Portnoy via AL Sonenshein |
| SRB1479 | *L. monocytogenes* 10403s *codY::spc* | D. Portnoy via AL Sonenshein |
| SRB1483 | LAC *codY*::φNΣ [erm::tet(M)] *lpdA*::φNΣ |  |
| SRB1522 | LAC *hla*::φNΣ |  |
| SRB1523 | LAC *codY*::φNΣ [*erm::tet*(M)] *hla*::φNΣ |  |
| SRB1702 | LAC Δ*sae* *codY*::φN𝚺 FL54a::pCL55 P*sae*P3*-saeRS* HOU0087:: *nuc-gfp* |  |
| SRB1708 | LAC pCK9 |  |
| SRB1709 | LAC *codY*::φNΣ pCK9 |  |
| SRB1712 | LAC *lpdA*::φNΣ |  |
| SRB1714 | LAC Δ*saePQRS-P23*-Δ*sae* pCL55 |  |
| SRB1715 | LAC Δ*sae* pCL55-P3*saeRS* *codY*::φNΣ |  |
| SRB1716 | LAC Δ*sae* pCL55 *codY*::φNΣ |  |
| SRB1885 | LAC *lpdA*::φNΣ pKM11 |  |
| SRB1935 | LAC *fakA(vrfB)*::φNΣ |  |
| SRB1936 | LAC *fakA(vrfB)*::φNΣ pKM11 |  |
| SRB2234 | LAC *lpdA*::φNΣ pAP1 pKM11 |  |
| SRB2344 | LAC *codY*::φNΣ [*erm::tet*(M)] *lpdA*::φNΣ pROKA |  |
| SRB2235 | LAC *codY*::φNΣ [erm::tet(M)] *lpdA*::φNΣ pAP3 |  |
| SRB2230 | LAC pROKA (empty) pKM11 | (11) |
| SRB2232 | LAC *lpdA*::φNΣ pROKA (empty) pKM11 |  |
| SRB2339 | LAC Δ*sae* pCL55 (empty) |  |
| SRB2340 | LAC *lpdA*::φNΣ Δ*sae* pCL55 (empty) |  |
| SRB2349 | LAC *lpdA*::φNΣpAP3 pKM11 |  |
| SRB2352 | COL pKM11 |  |
| SRB2353 | COL *lpdA*::φNΣ pKM11 |  |
| SRB2375 | LAC *Δsae* FL54a::pCL55 PsaeP3-saeRS *lpdA*::φNΣ |  |
| SRB2417 | LAC Δ*sae* pCL55_*saeRS* |  |
| SRB2418 | LAC *lpdA*::φNΣ Δ*sae* pCL55_*saeRS* |  |
| SRB2435 | LAC Δ*sae* pCL55_*saePQRS* |  |
| SRB2436 | LAC *lpdA*::φNΣ Δ*sae* pCL55_*saePQRS* |  |
| UK1 | *Clostridioides difficile* UK1 | D. Gerding via AL Sonenshein |
| 630E | *C. difficile* 630E (JIR8094) Ribotype 012, Em^S^ derivative of *C. difficile* 630 | (12) |
| LB-CD15 | *C. difficile* 630E *codY::ermC* | L. Bouillaut via AL Sonenshein |
| LB-CD16 | *C. difficile* UK1 *codY::ermC* | (13) |
|  | |  |

**References:**

1. Fey PD, Endres JL, Yajjala VK, Widhelm TJ, Boissy RJ, Bose JL, Bayles KW. 2013. A genetic resource for rapid and comprehensive phenotype screening of nonessential *Staphylococcus aureus* genes. mBio 4:e00537-00512.

2. Kreiswirth BN, Löfdahl S, Betley MJ, O’Reilly M, Schlievert PM, Bergdoll MS, Novick RP. 1983. The toxic shock syndrome exotoxin structural gene is not detectably transmitted by a prophage. 5936. Nature 305:709–712.

3. Gillaspy AF, Hickmon SG, Skinner RA, Thomas JR, Nelson CL, Smeltzer MS. 1995. Role of the accessory gene regulator (agr) in pathogenesis of staphylococcal osteomyelitis. Infect Immun 63:3373–3380.

4. Waters NR, Samuels DJ, Behera RK, Livny J, Rhee KY, Sadykov MR, Brinsmade SR. 2016. A spectrum of CodY activities drives metabolic reorganization and virulence gene expression in *Staphylococcus aureus*. Molecular Microbiology 101:495–514.

5. Kiedrowski MR, Kavanaugh JS, Malone CL, Mootz JM, Voyich JM, Smeltzer MS, Bayles KW, Horswill AR. 2011. Nuclease modulates biofilm formation in community-associated methicillin-resistant *Staphylococcus aureus*. PLoS One 6:e26714.

6. Mlynek KD, Bulock LL, Stone CJ, Curran LJ, Sadykov MR, Bayles KW, Brinsmade SR. 2020. Genetic and Biochemical Analysis of CodY-Mediated Cell Aggregation in *Staphylococcus aureus* Reveals an Interaction between Extracellular DNA and Polysaccharide in the Extracellular Matrix. Journal of Bacteriology 202:e00593-19.

7. Behera RK, Mlynek KD, Linz MS, Brinsmade SR. 2019. A Fluorescence-based Method to Study Bacterial Gene Regulation in Infected Tissues. JoVE (Journal of Visualized Experiments) e59055.

8. Mlynek KD, Sause WE, Moormeier DE, Sadykov MR, Hill KR, Torres VJ, Bayles KW, Brinsmade SR. 2018. Nutritional Regulation of the Sae Two-Component System by CodY in *Staphylococcus aureus*. Journal of Bacteriology 200:e00012-18.

9. Collins MM, Behera RK, Pallister KB, Evans TJ, Burroughs O, Flack C, Guerra FE, Pullman W, Cone B, Dankoff JG, Nygaard TK, Brinsmade SR, Voyich JM. 2020. The Accessory Gene saeP of the SaeR/S Two-Component Gene Regulatory System Impacts *Staphylococcus aureus* Virulence During Neutrophil Interaction. Frontiers in Microbiology 11:561.

10. Brinsmade SR, Kleijn RJ, Sauer U, Sonenshein AL. 2010. Regulation of CodY Activity through Modulation of Intracellular Branched-Chain Amino Acid Pools. J Bacteriol 192:6357–6368.

11. Jeong B, Shah MA, Roh E, Kim K, Park I, Bae T. 2022. *Staphylococcus aureus* does not synthesize arginine from proline under physiological conditions. bioRxiv https://doi.org/10.1101/2022.01.12.476138.

12. O’Connor JR, Lyras D, Farrow KA, Adams V, Powell DR, Hinds J, Cheung JK, Rood JI. 2006. Construction and analysis of chromosomal *Clostridium difficile* mutants. Molecular Microbiology 61:1335–1351.

13. Mooyottu S, Flock G, Kollanoor-Johny A, Upadhyaya I, Jayarao B, Venkitanarayanan K. 2015. Characterization of a multidrug resistant *C. difficile* meat isolate. Int J Food Microbiol 192:111–116.
