# Supplementary material for: Regulation of the Sae Two-Component System by Branched-Chain Fatty Acids in Staphylococcus aureus
Source: mBio. 2022 Sep 22;13(5):e01472-22. doi: 10.1128/mbio.01472-22 (PMC9600363; doi:10.1128/mbio.01472-22)
Supplement: TABLE S3 [file mbio.01472-22-s0008.docx]

|  | | |
| --- | --- | --- |
| **Oligo Name** | **Sequence (5' -> 3')** | **Purpose** |
| oAP05 | CATTCTCGAAAGGTTTACTAATGG | *fakA* Tn-confirm forward |
| oAP06 | CCAAAACGAACCATCATTTCAG | *fakA* Tn-confirm reverse |
| oAP19 | CACGTATAAGGCGTAATCATGGTCATAGCTG | pAP1 Gibson Construction Vector forward |
| oAP20 | AAAAATAGGACTGGCCGTCGTTTTACAAC | pAP1 Gibson Construction Vector reverse |
| oAP21 | CGGCCAGTCCTATTTTTGACTAAACCAAATGCTAACCCAGAAATACAAT | pAP1 Gibson Construction *sarA* P1 forward |
| oAP22 | TTCTCTGACATGTAATTCCTCCTTTTATAGATTATCTGGATCC | pAP1 Gibson Construction *sarA* P1 reverse |
| oAP23 | TGATTACGCCTTATACGTGAATAGCTCTACTTTCTGCTTTCAATCCTAATT | pAP1 Gibson Construction *lpdA* forward |
| oAP24 | GAGGAATTACATGTCAGAGAAACAATATGATTTAGTCGTTCT | pAP1 Gibson Construction *lpdA* reverse |
| oAP31 | GCTATGACCATGATTACGCCCTAATATATATTTGTATTTTCTAAAGTATACTGTTCGATACGCTG | pAP3 Gibson Construction BKD operon forward |
| oAP32 | GTAGAGCTATTCACGTATAAATAGGAGGATTTAACATGATTG | pAP3 Gibson Construction BKD operon reverse |
| oAP33 | AAAATACAAATATATATTAGGGCGTAATCATGGTCATAGCTGTTT | pAP3 Gibson Construction vector forward |
| oAP34 | ATCATGTTAAATCCTCCTATTTATACGTGAATAGCTCTACTTTCTGCTTTCAAT | pAP3 Gibson Construction vector reverse |
| oCS13 | AAAGAAGGTGTTGTGGCTGTTG | *lpdA* Tn-confirm forward |
| oCS14 | GGATGTGCGTGTGTTGTTAGT | *lpdA* Tn-confirm reverse |
| oSRB239 | GGATTGGCTTCACCTGAAAA | qRT-PCR for *rpoC* |
| oSRB240 | CTTTCACGACGTACTTTAGA | qRT-PCR for *rpoC* |
| oWY25 | GGGACAGCTGATAGAAAC | pROKA sequencing forward |
| oWY26 | GGATATCTATTTATTCCG | pROKA sequencing reverse |
| oWY33 | CCACCTGACGTCTAAGAAACC | pCL55 sequencing forward |
| oWY34 | CGTAGTATCTATACTTCGTTTC | pCL55 sequencing reverse |
| oWY45 | GGCGATAAACGTGTCGAGTT | qRT-PCR for *walK* |
| oWY46 | GCGCTTCCACAATCTCTTTC | qRT-PCR for *walK* |
| oWY47 | GTGACCGCTACAAAACGTGA | qRT-PCR for *hptS* |
| oWY48 | TTAAACGTGGGGTGTTAGCC | qRT-PCR for *hptS* |
| oWY51 | GTGGAACGTCAACTTGCAGA | qRT-PCR for *lytS* |
| oWY52 | TCTCGTGCCTTTTCGCTATT | qRT-PCR for *lytS* |
| oWY59 | TGAAATCTCGCATGAACTGG | qRT-PCR for *graS* |
| oWY60 | AAATGGCGTTTCCGCTAAAT | qRT-PCR for *graS* |
| oWY61 | AAACGGTGAAACTGTTGAAGG | qRT-PCR for *saeP* |
| oWY62 | CGTAGTCAACCATTGCGATTT | qRT-PCR for *saeP* |
| oWY71 | TCATTGTCTTTTCGTTGTCG | qRT-PCR for *desK* |
| oWY72 | GTAAATGGAACGGCAAAAGC | qRT-PCR for *desK* |
| oWY77 | ACCGTTATTTTGACCGCGTA | qRT-PCR for *arlS* |
| oWY78 | GCCAGCGCAATGATATACAA | qRT-PCR for *arlS* |
| oWY81 | GAGCCGGCTAAATAGTGTCG | qRT-PCR for *srrB* |
| oWY82 | TCATCAACCCACCAGGATTT | qRT-PCR for *srrB* |
| oWY83 | TTGTCGCATTTGATGGTGAT | qRT-PCR for *phoP* |
| oWY84 | CGGGCAACAACTTCTCTAGG | qRT-PCR for *phoP* |
| oWY87 | ATCGGGTCAAGAACAATTCG | qRT-PCR for *airS* |
| oWY88 | CGCAGCATGTGCTGTTAGTT | qRT-PCR for *airS* |
| oWY93 | GCATGCTAGCTGCATTTCTG | qRT-PCR for *vraS* |
| oWY94 | GCGAGTACCGAACCAACAAT | qRT-PCR for *vraS* |
| oWY99 | AGATGACATGCCTGGCCTAC | qRT-PCR for *agrC* |
| oWY100 | CGGAAAATTCATTTCTTGTGC | qRT-PCR for *agrC* |
| oWY103 | GTGGCAATTAGTGGGAGCAT | qRT-PCR for *kdpD* |
| oWY104 | ATCCTAATGCAACGGTTTGG | qRT-PCR for *kdpD* |
| oWY109 | TTGCTGCTGATGAAAAGTCG | qRT-PCR for *hssS* |
| oWY110 | GTTGCAAAGCGATGTCAATG | qRT-PCR for *hssS* |
| oWY113 | TTGCAATGAATGATGCTGCT | qRT-PCR for *nreB* |
| oWY114 | GCATGAATTCCCCTGTCTTG | qRT-PCR for *nreB* |
| oWY119 | TCATTCGCCCAATTATTATGAA | qRT-PCR for *braS*(*nsaS*) |
| oWY120 | TTAGGCAAGTCCGCTTCACT | qRT-PCR for *braS*(*nsaS*) |
| oWY123 | CAAATGATCACAGCATTTGGTACAG | qRT-PCR for *gyrB* |
| oWY124 | CGGCATCAGTCATAATGACGAT | qRT-PCR for *gyrB* |
