# Supplementary material for: Regulation of the Sae Two-Component System by Branched-Chain Fatty Acids in Staphylococcus aureus
Source: mBio. 2022 Sep 22;13(5):e01472-22. doi: 10.1128/mbio.01472-22 (PMC9600363; doi:10.1128/mbio.01472-22)
Supplement: TABLE S4 [file mbio.01472-22-s0009.docx]

|  | | |
| --- | --- | --- |
| **Plasmid ID** | **Genotype/properties** | **Reference** |
| pAP1 | *sarA* P1 promoter driving *lpdA* expression in pROKA, Km^R^ |  |
| pAP3 | sarA P1 promoter driving *lpdA, bkdA1, bkdA2, and bkdB* in pROKA, Km^R^ |  |
| pCL55 | Empty vector control, Ap^R^, Cm^R^ | (1) |
| pCL55_Sae | Integrated vector containing *saePQRS* strain USA300 | (2) |
| pCL55_saeRS | Integrated vector containing *saeRS* with P3 promoter | (2) |
| pKM11 | GFP under *sae* P1 promoter control | (3) |
| pKM26 | *sarA* P1 promoter driving *icaB* expression, Em^R^ | (3) |
| pROKA | Empty vector control for pAP1 and pAP3, Km^R^ | (4) |

**Supplemental References:**

1. Lee CY, Buranen SL, Ye ZH. 1991. Construction of single-copy integration vectors for *Staphylococcus aureus*. Gene 103:101–105.

2. Jeong D-W, Cho H, Lee H, Li C, Garza J, Fried M, Bae T. 2011. Identification of the P3 Promoter and Distinct Roles of the Two Promoters of the SaeRS Two-Component System in *Staphylococcus aureus*. Journal of Bacteriology 193:4672–4684.

3. Mlynek KD, Bulock LL, Stone CJ, Curran LJ, Sadykov MR, Bayles KW, Brinsmade SR. 2020. Genetic and Biochemical Analysis of CodY-Mediated Cell Aggregation in *Staphylococcus aureus* Reveals an Interaction between Extracellular DNA and Polysaccharide in the Extracellular Matrix. Journal of Bacteriology 202:e00593-19.

4. Jeong B, Shah MA, Roh E, Kim K, Park I, Bae T. 2022. *Staphylococcus aureus* does not synthesize arginine from proline under physiological conditions. bioRxiv https://doi.org/10.1101/2022.01.12.476138.
